# Integrative Cell Type-Specific Multi-Omics Approaches Reveal Impaired Programs of Glial Cell Differentiation in Mouse Culture Models of DM1

**DOI:** 10.3389/fncel.2021.662035

**Published:** 2021-05-05

**Authors:** Anchel González-Barriga, Louison Lallemant, Diana M. Dincã, Sandra O. Braz, Hélène Polvèche, Paul Magneron, Cédric Pionneau, Aline Huguet-Lachon, Jean-Baptiste Claude, Cerina Chhuon, Ida Chiara Guerrera, Cyril F. Bourgeois, Didier Auboeuf, Geneviève Gourdon, Mário Gomes-Pereira

**Affiliations:** ^1^Sorbonne Université, Inserm, Institut de Myologie, Centre de Recherche en Myologie, Paris, France; ^2^Inserm UMR 1163, Institut Imagine, Université Paris Cité, Paris, France; ^3^Laboratory of Biology and Modeling of the Cell, Université de Lyon, ENS de Lyon, Université Claude Bernard, CNRS UMR 5239, Inserm U1210, Lyon, France; ^4^Inserm/UEVE UMR 861, Université Paris Saclay I-STEM, Corbeil-Essonnes, France; ^5^Sorbonne Université, Inserm, UMS PASS, Plateforme Post-génomique de la Pitié Salpêtrière (P3S), Paris, France; ^6^Proteomics Platform Necker, Université de Paris - Structure Fédérative de Recherche Necker, Inserm US24/CNRS UMS 3633, Paris, France

**Keywords:** myotonic dystrophy, neurons, astrocytes, oligodendrocytes, transcriptomics, phosphoproteomics, splicing, ontology

## Abstract

Myotonic dystrophy type 1 (DM1) is a neuromuscular disorder caused by a non-coding CTG repeat expansion in the *DMPK* gene. This mutation generates a toxic CUG RNA that interferes with the RNA processing of target genes in multiple tissues. Despite debilitating neurological impairment, the pathophysiological cascade of molecular and cellular events in the central nervous system (CNS) has been less extensively characterized than the molecular pathogenesis of muscle/cardiac dysfunction. Particularly, the contribution of different cell types to DM1 brain disease is not clearly understood. We first used transcriptomics to compare the impact of expanded CUG RNA on the transcriptome of primary neurons, astrocytes and oligodendrocytes derived from DMSXL mice, a transgenic model of DM1. RNA sequencing revealed more frequent expression and splicing changes in glia than neuronal cells. In particular, primary DMSXL oligodendrocytes showed the highest number of transcripts differentially expressed, while DMSXL astrocytes displayed the most severe splicing dysregulation. Interestingly, the expression and splicing defects of DMSXL glia recreated molecular signatures suggestive of impaired cell differentiation: while DMSXL oligodendrocytes failed to upregulate a subset of genes that are naturally activated during the oligodendroglia differentiation, a significant proportion of missplicing events in DMSXL oligodendrocytes and astrocytes increased the expression of RNA isoforms typical of precursor cell stages. Together these data suggest that expanded CUG RNA in glial cells affects preferentially differentiation-regulated molecular events. This hypothesis was corroborated by gene ontology (GO) analyses, which revealed an enrichment for biological processes and cellular components with critical roles during cell differentiation. Finally, we combined exon ontology with phosphoproteomics and cell imaging to explore the functional impact of CUG-associated spliceopathy on downstream protein metabolism. Changes in phosphorylation, protein isoform expression and intracellular localization in DMSXL astrocytes demonstrate the far-reaching impact of the DM1 repeat expansion on cell metabolism. Our multi-omics approaches provide insight into the mechanisms of CUG RNA toxicity in the CNS with cell type resolution, and support the priority for future research on non-neuronal mechanisms and proteomic changes in DM1 brain disease.

## Introduction

Myotonic dystrophy type 1 (DM1) is a life-threatening rare disease presenting great variability in clinical manifestations and age of onset (Ashizawa and Sarkar, 2011; Harper, 2001; Udd and Krahe, 2012). Overall, DM1 has a prevalence of 1/3,000-1/8,000 individuals worldwide (Ashizawa et al., 2018), making it the most common muscular dystrophy in adults. Peripheral clinical features of DM1 include muscle weakness, myotonia, cardiac conduction abnormalities, premature cataracts and abnormal insulin metabolism. Central nervous system (CNS) impairment is also prominent, particularly in the congenital cases that present varying degrees of intellectual disability, attention-deficit hyperactivity disorder and autism spectrum disorder (Johnson et al., 2019). The cognitive profile of infantile, juvenile and adult patients is characterized by alterations in multiple neurological domains, such as global and social cognition, memory, language, executive dysfunction and visuospatial deficits. Psychiatric and emotional problems involve apathy, anxiety, avoidant behavior, and excessive daytime sleepiness (Meola and Sansone, 2007; Okkersen et al., 2017a). The wide and heterogeneous cognitive and neuropsychological profile in DM1 suggests the involvement of multiple brain areas and neuronal circuits. This hypothesis has been corroborated by functional studies reporting DM1 changes in the connectivity of brain networks (Serra et al., 2014), and studies of structural neuroimaging revealing widespread white matter changes and a decrease in gray matter volume in multiple brain regions (Gourdon and Meola, 2017; Okkersen et al., 2017b; Minnerop et al., 2018).

DM1 is caused by an expansion of a CTG trinucleotide repeat sequence in the 3’-UTR of the *DMPK* gene (Aslanidis et al., 1992; Brook et al., 1992; Fu et al., 1992; Mahadevan et al., 1992). This repeat contains less than 38 units in the non-affected population. A repeat length above ∼50 CTG units results in DM1 manifestations, with an age of onset and severity that correlate with the expansion size: longer repeats are associated with more severe clinical forms and earlier ages of onset (De Antonio et al., 2016). DM1 was the first disease ever described for which the underlying molecular pathogenesis involves a toxic RNA gain-of-function (Day and Ranum, 2005). Similar mechanisms of RNA toxicity were later unveiled in other disorders (Sicot et al., 2011; Sicot and Gomes-Pereira, 2013; Neueder, 2019). In DM1, expanded CUG repeats in *DMPK* mRNA form stable hairpin structures that are retained in the cell nucleus, where they form large aggregates (RNA foci) by abnormal binding of ribonuclear proteins. Some important factors involved in RNA metabolism, like the muscleblind-like (MBNL) family of splicing regulators, are sequestered by RNA foci (Miller et al., 2000; Fardaei et al., 2002). Several studies have shown that the limited availability of functional MBNL1, MBNL2, and/or MBNL3 contributes considerably to the development of molecular features of DM1 (Osborne et al., 2009; Du et al., 2010; Batra et al., 2014; Goodwin et al., 2015), as well as relevant phenotypes in mice (Kanadia et al., 2003; Matynia et al., 2010; Charizanis et al., 2012; Lee et al., 2013; Choi et al., 2015; Dixon et al., 2015; Thomas et al., 2017). In addition, stress responses triggered by the expression of mutant *DMPK* RNA cause the stabilization/activation of MBNL antagonists, like CELF1 (Kuyumcu-Martinez et al., 2007; Wang et al., 2007), STAU1 (Ravel-Chapuis et al., 2012), and hnRNPA1 (Li et al., 2020). Together, these proteins function as developmental regulators of alternative splicing, and their imbalance in DM1 causes primarily the aberrant persistence of fetal splicing isoforms in adult tissues, as well as other pre-mRNA processing defects. This pathological mechanism has been termed “spliceopathy” and affects hundreds of genes in several tissues and organs, many of which have been directly related to some peripheral manifestations of DM1. For example, myotonia is mediated by splicing defects in *CLCN1*; muscle weakness is likely the result of multiple missplicing events (*BIN1, CACNA1S DMD*, and *RYR1*); cardiac conduction defects may also be the consequence of at least two reported splicing abnormalities (*SCN5A*, and *TNNT2*) (Sznajder and Swanson, 2019).

In the brain, *DMPK* is expressed in neuronal and non-neuronal cell types (Zhang et al., 2016), resulting in accumulation of RNA foci in neurons, astrocytes and oligodendrocytes (Jiang et al., 2004; Hernández-Hernández et al., 2013; Sicot et al., 2017). MBNL sequestration has been reported as a modulator of RNA toxicity and disease pathogenesis in the brain (Matynia et al., 2010; Charizanis et al., 2012; Suenaga et al., 2012; Goodwin et al., 2015; Wang et al., 2017, 2018; Lee et al., 2019). In addition, CELF proteins were also suggested to contribute to CNS pathogenesis (Dhaenens et al., 2011; Hernández-Hernández et al., 2013). Still, the molecular and cellular mechanisms driving DM1 neurological impairment remain largely unknown. Previous studies analyzed the transcriptome of DM1 patients to uncover disease-related alterations (Batra et al., 2014; Goodwin et al., 2015; Otero et al., 2021). While many of the events found are specific to the CNS, an inherent limitation of studying tissue samples is the lack of cell type resolution. In particular, we do not know the extent to which different cell types are affected in the brain, and their contribution to the neuropsychological symptoms. To completely understand brain disease mechanisms and develop efficient therapies, a thorough analysis of cell type-specific events is indispensable. Primary brain cells generated efficiently in culture can obviate the technical limitations with the use of cell populations acutely isolated from fresh tissue, providing suitable model systems for studying disease pathogenesis with cell type resolution.

Here, we sought to investigate the molecular and cellular mechanisms perturbed by the CTG expansion in a cell-specific manner, using a transgenic mouse model of DM1 as a renewable source of homogenous primary cultures of neurons, astrocytes and oligodendrocytes. DMSXL mice carry a transgenic fragment of the human *DM1* locus containing more than 1,000 CTG trinucleotides (Gomes-Pereira et al., 2007). These animals display key molecular hallmarks of DM1, including ribonuclear foci and RNA missplicing in multiple tissues, as well as relevant muscle, cardiac and respiratory phenotypes (Huguet et al., 2012; Panaite et al., 2013; Algalarrondo et al., 2015). Importantly for our study, the expression of expanded CUG-containing *DMPK* transcripts in the CNS causes RNA foci accumulation in DMSXL neurons and glial cells (Hernández-Hernández et al., 2013; Sicot et al., 2017). The dysregulation of synaptic and glial proteins in these mice suggests underlying problems in multiple brain cell types. In particular, data implicating Bergmann glia dysfunction in the cerebellum-dependent motor incoordination of DMSXL mice (Sicot et al., 2017), further underscore the increasing importance of exploring the involvement on non-neuronal cells. The complexity of cell type composition in the brain, prompted us to use homogenous primary cultures derived from DMSXL mice to perform an integrative and cell type-resolved multi-omics approach, to investigate changes in the transcriptome and phosphoproteome of the major brain cell types, and explore the downstream functional consequences at single cell level.

## Materials and Methods

### Transgenic Mice

DMSXL transgenic mice (>99.8% C57BL/6 background) carry 45 kb of human genomic DNA from a DM1 patient (Seznec et al., 2000; Gomes-Pereira et al., 2007) within the first intron of the *Fbxl7* gene (data not shown). The breeding of hemizygous DMSXL mice generated DMSXL homozygotes and WT controls in the same litter. The DMSXL mice used in this study were homozygous for the human transgene, and carried more than 1,500 CTG repeats within the human *DMPK* gene. Transgenic DMSXL status was assessed by multiplex PCR using the oligonucleotide primers, which hybridize the *DMPK* transgene and the transgene integration site in the mouse *Fbxl7* gene: FBF (forward, TCCTCAGAAGCACTCATCCG), FBFBR (reverse, AACCCTGTATTTGACCCCAG) and FBWDR (reverse, ACCTCCATCCTTTCAGCACC). FBF and FBFBR primers hybridize the mouse *Fbxl7* gene, amplifying a sequence of 167 bp in WT mice. FBWDR primer hybridizes specifically the human transgene, generating a 236 bp PCR product.

### Cell Culture

Primary dissociated cell cultures of mouse cortical neurons, astrocytes, and oligodendrocytes were produced as previously described (Braz et al., 2020). Astrocyte precursors were extracted by magnetic cell sorting with magnetic anti-ACSA-2 microbeads (Miltenyi, 130-097-678), following the manufacturer’s instructions. The purity of primary cell cultures was estimated by immunofluorescence of cell type-specific protein markers: neurons, TUBB3; OPC, OLIG2; OL, MBP; Astrocytes, GFAP (Braz et al., 2020).

### RNA Isolation

RNA extraction was performed with the RNeasy Mini Kit (Qiagen, 74104) following the manufacturer’s protocol, with an additional DNAse digestion step (RNase-Free DNase Set, Qiagen, 79254) after the first wash with RW1 buffer. RNA concentration was assessed using a NanoDrop (Thermo Fisher Scientific).

### RNA-Seq

RNA quality of samples from primary cells (*n* = 4 independent cultures per cell type, per genotype) was verified by electrophoresis on an agarose gel. Illumina-compatible precapture barcoded mRNA libraries were constructed, and a series of 32 barcoded libraries was pooled at equimolar concentrations. The capture process was performed according to the manufacturer’s protocols for TruSeq Stranded mRNA (Illumina) and sequenced on an Illumina HiSeq2500, with a mean read depth of 49.6E6 ± 1.3E6 per sample (minimum, 35.3E6 reads per sample; maximum, 65.9E6). Global gene expression was analyzed as previously described (Lambert et al., 2018), using the DESeq2 package. Significant expression changes were considered for further analysis if absolute fold change between genotypes > 1.4, and *p*-value (corrected for multiple comparisons) < 0.05. Alternative splicing analyses were performed using the open source FaRLine pipeline (Benoit-Pilven et al., 2018). Significant missplicing events were selected for further analysis if the percentage of splicing inclusion (PSI) between genotypes was > 10% with *p*-value (corrected for multiple comparisons) < 0.05 (Fisher’s test). PSI was calculated as: (inclusion of alternative exon)/[(inclusion of alternative exon) + (exclusion of alternative exon)]. The datasets generated for this study can be found in the Gene Expression Omnibus repository, https://www.ncbi.nlm.nih.gov/geo/query/acc.cgi?&acc=GSE162093.

### Reverse Transcription and PCR Assays

For reverse transcription, 150–500 ng RNA was subjected to cDNA synthesis using the SuperScript II first-strand synthesis system (Thermo Fisher Scientific) with random hexamer primers in a total volume of 20 μL. In RT(−) control samples, water was added instead of reverse transcriptase.

Primer sets for PCR were designed using MacVector and NCBI Primer-Blast software. Amplification products were resolved by agarose gels electrophoresis. Primer sequences are listed in Supplementary Table 1.

#### Semi-Quantitative PCR

One μL of cDNA was used in PCR amplifications according to standard procedures. Cycling conditions were optimized for each target independently in pilot experiments. PCR products were analyzed on 1.5–2.5% agarose gels stained by ethidium bromide. Quantification of signals was done using the Image Lab software (Bio-Rad). PSI was calculated for each alternative exon.

#### Real-Time qPCR

For real-time RT-PCR (RT-qPCR), all procedures were performed following the Minimum Information for Publication of Quantitative Real-Time PCR Experiments (MIQE) guidelines (Bustin et al., 2009). Amplification efficiency of primer sets was determined using standard curves in each qPCR plate. Sample preparation and cycling conditions were done as described elsewhere (Huguet et al., 2012; Hernández-Hernández et al., 2013). All samples were normalized relative to the expression of an internal control: RNA Polymerase II Subunit A (*Polr2a*).

### Protein Isolation

Total protein from primary cells was extracted using RIPA buffer (Thermo Fisher Scientific, 89901) supplemented with 0.05% CHAPS (Sigma, C3023), 1× complete protease inhibitor (Sigma-Aldrich, 04693124001) and 1× PhosSTOP phosphatase inhibitor (Sigma-Aldrich, 04906845001). Protein concentrations were determined using the Pierce BCA Protein Assay Kit (Thermo Fisher Scientific, 23227).

### Mass Spectrometry

#### Protein Digestion and Phosphopeptide Enrichment by Titanium Dioxide (TiO_2_)

Proteins were first reduced with 0.1 M dithiothreitol (DTT) final concentration at 60°C for 1 h. Then, proteins were digested using a FASP method (filter aided sample preparation) according to Lipecka et al. (2016). Phosphopeptide enrichments were carried out using Titansphere TiO_2_ Spin tips (3 mg/200 μL, Titansphere PHOS-TiO, GL Sciences Inc.) on the digested proteins for each biological replicate. Briefly, the TiO_2_ Spin tips were conditioned with 20 μL of solution A (80% acetonitrile, 0.4% TFA), centrifuged at 3,000 g for 2 min and equilibrated with 20 μL of solution B (75% acetonitrile, 0.3% TFA, 25% lactic acid) followed by centrifugation at 3,000 g for 2 min. Peptides were dissolved in 20 μL of solution A, mixed with 100 μL of solution B and centrifuged at 1,000 g for 10 min. Sample was applied back to the TiO_2_ Spin tips twice more in order to increase the adsorption of the phosphopeptides to the TiO_2_. Spin tips were washed sequentially with 20 μL of solution B and twice with 20 μL of solution A. Phosphopeptides were eluted by the sequential addition of 50 μL of 5% NH_4_OH and 50 μL of 5% pyrrolidine. Centrifugation was carried out at 1,000 g for 5 min. Phosphopeptides were purified using GC Spin tips (GL-Tip, Titansphere, GL Sciences Inc.). Briefly, the GC Spin tips were conditioned according to manufacturer instruction, then eluted phosphopeptides from the TiO_2_ Spin tips were added to the GC Spin tips and centrifuged at 1,000 g for 5 min. GC Spin tips were washed with 20 μL of 0.1% TFA in HPLC-grade water solution C. Phosphopeptides were eluted with 70 μL of 80% acetonitrile, 0.1% TFA solution A (1,000 g for 5 min) and vacuum dried.

#### NanoLC-MS/MS Protein Identification and Quantification

Samples were resuspended in 35 μL of 0.1% TFA in HPLC-grade water. For each run, 5 μL was injected in a nanoRSLC-Q Exactive PLUS (RSLC Ultimate 3000, Thermo Fisher Scientific). Phosphopeptides were loaded onto a μ-precolumn (Acclaim PepMap 100 C18, cartridge, 300 μm i.d. × 5 mm, 5 μm, Thermo Fisher Scientific) and were separated on a 50 cm reversed-phase liquid chromatographic column (0.075 mm ID, Acclaim PepMap 100, C18, 2 μm, Thermo Fisher Scientific). Chromatography solvents were (A) 0.1% formic acid in water, and (B) 80% acetonitrile, 0.08% formic acid. Phosphopeptides were eluted from the column with the following gradient 5–40% B (120 min), 40–80% (6 min). At 127 min, the gradient returned to 5% to re-equilibrate the column for 20 min before the next injection. Two blanks were run between each sample to prevent sample carryover. Phosphopeptides eluting from the column were analyzed by data dependent MS/MS, using top-8 acquisition method. Phosphopeptides were fragmented using higher-energy collisional dissociation (HCD). Briefly, the instrument settings were as follows: resolution was set to 70,000 for MS scans and 17,500 for the data dependent MS/MS scans in order to increase speed. The MS AGC target was set to 1 × 10^6^ counts with maximum injection time set to 200 ms, while MS/MS AGC target was set to 2 × 10^5^ with maximum injection time set to 120 ms. The MS scan range was from 400 to 1,800 m/z. MS and MS/MS scans were recorded in profile mode. Dynamic exclusion was set to 30 s.

#### Data Processing Following NanoLC-MS/MS Acquisition

The MS files were processed with the MaxQuant software version 1.5.8.3 and searched with Andromeda search engine against the UniProtKB/Swiss-Prot *Mus musculus* database (release 27-07-2017, 16923 entries). To search parent mass and fragment ions, we set an initial mass deviation of 4.5 ppm and 0.5 Da, respectively. The minimum peptide length was set to seven amino acids and strict specificity for trypsin cleavage was required, allowing up to two missed cleavage sites. Carbamidomethylation (Cys) was set as fixed modification, whereas oxidation (Met), N-term acetylation and phosphorylation (Ser, Thr, Tyr) were set as variable modifications. The match between runs option was enabled with a match time window of 0.7 min and an alignment time window of 20 min. The false discovery rates (FDRs) at the protein and peptide level were set to 1%. Scores were calculated in MaxQuant as described previously (Cox and Mann, 2008). The reverse and common contaminants hits were removed from MaxQuant output. The phosphopeptides output table and the corresponding logarithmic intensities were used for subsequent statistical analysis using the Perseus software platform. Significant phosphorylation changes were considered for further analysis if the phosphosite was detected only in all the samples of one of the genotypes (but in none of the other) or when fold change between genotypes > 1.4 and *p*-value (corrected for multiple comparisons) < 0.05. The datasets generated for this study can be found in the PRIDE repository: http://www.ebi.ac.uk/pride/archive/projects/PXD025011.

### Western Blot

Ten micrograms of total protein were mixed with 2X Laemmli Sample Buffer (Sigma-Aldrich, S3401), denatured for 5 min at 95°C and resolved in gradient (4–20%) Mini-PROTEAN TGX Stain-Free polyacrylamide gels (Bio-Rad, 4568096). After electrophoresis, gels were activated for 45 s under UV light, proteins were transferred onto nitrocellulose membranes using Trans-Blot Turbo Transfer System (Bio-Rad, 1704150) and total protein on the membrane was imaged using the ChemiDoc MP Imaging System (Bio-Rad, 17001402). Membranes were then blocked in 5% Blotto non-fat dry milk (Santa Cruz Biotech, sc2325) in 1× TBS-T (10 mM Tris-HCl, 0.15 M NaCl, 0.05% Tween 20) over night at 4°C. Incubations with either 1/500 anti-CAPZB (GeneTex, GTX89785) or 1/1,000 anti-PALM (Abcam, ab234739) primary antibodies were performed at room temperature for 3 h. Then, after three washes in TBS-T, membranes were incubated with 1/5,000 dilution of the following secondary antibodies conjugated to horseradish peroxidase (HRP) for 1 h at room temperature: donkey anti-goat IgG (Jackson ImmunoResearch, 705-035-147) for CAPZB membrane and goat anti-rabbit IgG (Thermo Fisher Scientific, 31460) for PALM membrane. Membranes were washed three times, developed by Clarity Western ECL Substrate (Bio-Rad, 1705061) and imaged with the ChemiDoc MP Imaging System (Bio-Rad, 17001402). Band intensity was quantified using Image Lab Software (Bio-Rad).

### Two-Dimensional Western Blot

#### Sample Preparation and Labeling

Total protein lysates were extracted from cells in a buffer composed of 7 M urea, 2 M thiourea, 1% CHAPS, 10% isopropanol, 10% isobutanol, 0.5% Triton X-100 and 0.5% SB3-10. Proteins were precipitated using the Perfect Focus kit (G-Biosciences) and pellets were resuspended in the same buffer. The protein content was assessed by the Quick Start^TM^ Bradford protein assay (BioRad) using BSA as standard. For each sample, 15 μg of proteins were supplemented with 20 mM Tris-HCl (pH 8.8), labeled with 120 pmol of Cy3 (Fluoprobes) by incubation during 30 min on ice and then quenched with 0.35 mM lysine for 10 min. Then, unlabeled proteins were added to corresponding Cy3-labeled proteins, for a total of 94 μg for WT1 and DMSXL1 samples (15 μg of Cy3-labeled + 79 μg of unlabeled) and for a total of 44 μg for WT2 and DMSXL2 samples (15 μg of Cy3-labeled + 29 μg unlabeled).

#### Two-Dimensional Difference Gel Electrophoresis

Two-dimensional electrophoresis was performed on 7-cm gels with pH ranges 4–7 using commercial strips (Bio-Rad). Strips were passively rehydrated overnight directly with the samples diluted in a rehydration buffer (Urea 7 M; Thiourea 2 M; CHAPS 1%; SD3-10 0.5%; Triton-X100 0.5%; Isobutanol 10%) supplemented with 40 mM DTT and 0.5% IPG Buffer 4–7 (Cytiva). IEF migration was set as follows: 1.5 h at 50 V, 1 h at 200 V, 45 min gradient from 200 to 1,000 V, 45 min at 1,000 V, 1 h gradient from 1,000 to 4,000 V, 3 h at 4,000 V, for a total of 16,000 VHrs. Following IEF, strips were incubated again in the corresponding rehydration buffer for 2 h and a second step of IEF migration was carried out: 30 min at 200 V, 1 h gradient from 200 V to 4,000 V, 3 h 45 at 4,000 V, for a total of 11,000 VHrs. Strips were incubated for 15 min in equilibration buffer (Urea 6 M, Tris pH 8.8 75 mM, Glycerol 26%, SDS 2%) supplemented with 65 mM DTT then 15 min in equilibration buffer supplemented with 135 mM iodoacetamide. The second dimension was performed in 10% acrylamide gels in a Tris-Glycine-SDS buffer. Current was set at 10 mA per gel during 1.5 h then at 25 mA per gel. Gel and membranes images were acquired on a scanner Ettan DIGE Imager (GE Healthcare).

#### Transfer, Blotting, and Image Acquisition

Proteins were transferred onto nitrocellulose membranes using Trans-Blot Transfer System (Bio-Rad) and total protein on the membrane was imaged using the ChemiDoc Imaging System (Bio-Rad). Membranes were blocked in 5% Blotto non-fat dry milk (Santa Cruz Biotech, sc2325) in 1× TBS-T (10 mM Tris-HCl, 0.15 M NaCl, 0.05% Tween 20) over night at 4°C. Incubation with 1/500 goat anti-CAPZB (GeneTex, GTX89785) and 1/1,000 rabbit anti-PALM (Abcam, ab234739) primary antibodies was performed at room temperature for 2 h and then washed three times in TBS-T. Membranes were incubated first with 1/15,000 680RD donkey anti-rabbit (LI-COR Biosciences, 925-68073) for 1 h at room temperature, washed three times and imaged using the Odyssey^®^ CLx Imaging System (LI-COR) to detect PALM signal. To detect CAPZB signal, we incubated the membranes with 1/5,000 donkey anti-goat IgG conjugated to HRP (Jackson ImmunoResearch, 705-035-147), washed three times and develop using the Clarity Western ECL Substrate (Bio-Rad, 1705061) in the ChemiDoc MP Imaging System (Bio-Rad, 17001402).

### Immunocytochemistry

#### Fluorescent *in situ* Hybridization (FISH) and Immunofluorescence Analysis (IFA)

Primary cells were fixed after 14 days *in vitro* for 15 min in 4% PFA (VWR, J61899.AP) and ribonuclear inclusions were detected using a 5′-Cy3-labeled (CAG)_5_ PNA probe, as previously described (Huguet et al., 2012). IFA combined with FISH was performed as previously described (Hernández-Hernández et al., 2013). The following primary antibodies were diluted as specified by the manufacturer: TUBB3 (Covance, PRB-435P), OLIG2 (Merck, AB9610), MBP (Abcam, ab40390), GFAP (Dako cytomation, Z0334), INF2 (ProteinTech, 20466-1-A), NUMA (GeneTex, GTX64368), SORBS1 (Proteintech, 13854-1-AP), and CLASP1 (Abcam, ab108620).

#### Microscope Imaging and Image Analysis

Images were acquired with a Zeiss ApoTome 2 fluorescent microscope or a Leica SP8 SMD confocal microscope (×63 and ×40 objectives). RNA foci were counted in 3D stacks using the Spot Detector plugin of the ICY bioimageanalysis open-source program^1^. INF2 and NUMA nucleo-cytoplasmic ratio was quantified on Z projections (using maximal intensity) of Z-stack confocal images, by quantifying the ratio between mean gray intensity values in nucleus and cytoplasm. IF and FISH images were treated with Fiji—ImageJ software (Schindelin et al., 2012) to create the figures.

### Statistics and Ontology Analyses

Statistical analyses were performed with Prism (GraphPad Software, Inc.). Data are represented as mean ± standard error of mean (SEM). After performing a normality test on the numeric variables, we used two-tailed Student’s *t*-test for parametric data and Mann-Whitney *U* test for non-parametric data, when two groups were compared. The statistical tests performed in each quantification are detailed in the corresponding figure legend. To calculate the statistical significance of the overlap between two groups of genes we used an online tool^2^. This statistical test calculates the probability (*p-*value) of finding the computed overlap if the genes were present at random in both groups, as well as the representation factor: fold-change between overlap observed and number expected by chance (Kim et al., 2001). Gene ontology (GO) analysis, as well as transcription and miRNA binding site enrichment were performed using Webgestalt tool^3^, using the total transcripts or proteins detected as background. Kinase enrichment analysis (KEA) was performed using the Enrichr suite of gene list enrichment tools^4^. *p*-values were Benjamin-Hochberg corrected (FDR).

## Results

### Characterization of Brain Cell Types From the DMSXL Mouse Model

#### Isolation of Neurons, Glial Cells, and Their Precursors

**FIGURE 1:**
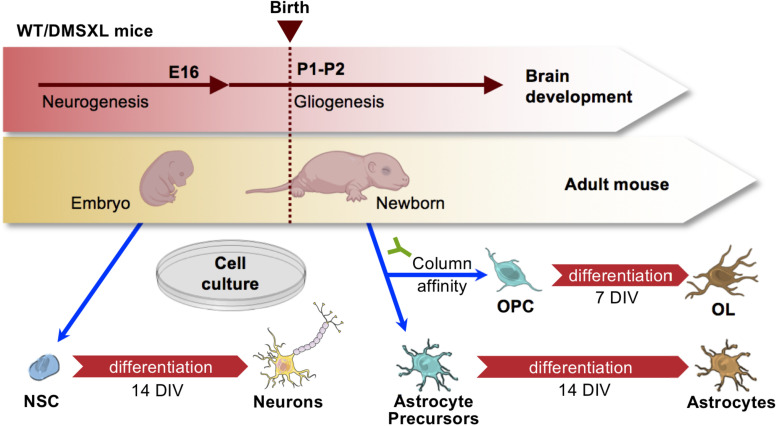
Strategy for the purification of primary brain cells from mouse cortex. Schematic representation of brain cell isolation from embryos and newborns. Precursor NSC were isolated from embryos at E16, prior to gliogenesis onset, and subsequently differentiated for 14 days *in vitro* (DIV). Astrocytes and OPC were isolated at birth. Astrocytes were differentiated for 14 DIV, while OPC were differentiated into OL over 7 DIV. Drawings of mouse embryo and newborn created with BioRender.com. Drawings of mouse embryo and newborn created with BioRender.com. Drawings of brain cells from Servier Medical Art, licensed under a Creative Commons Attribution 3.0 Unported License (CC BY 3.0).

To investigate DM1-associated brain molecular pathogenesis with cell type resolution, we established homogenous primary cultures of individual cell types of the DMSXL mouse brain. To this end, we followed a primary cell isolation strategy from mouse brain cortices (Figure 1), which allowed us to obtain cell cultures of enough purity to study intrinsic molecular changes in neurons, astrocytes and oligodendrocytes (OL), with minimal cross contamination between cell types. Neuron stem cells (NSC) were collected from E16 embryos, at a stage of brain development prior to gliogenesis, and were then differentiated in culture. Oligodendrocyte precursor cells (OPCs) were collected from newborn pups, by column affinity isolation methods of cell sorting, using magnetic microbeads conjugated to anti-CD140a antibodies. A portion of the OPC population was collected for analysis after growing them in proliferation medium for 7 days *in vitro* (DIV), while the remaining cells were cultured in differentiation medium for an additional 7 DIV, to obtain matched differentiated OL from the same progenitors. The column flow-through, containing mainly astrocytes, was cultured for 14 DIV in astrocyte medium, to further enrich this cell type, eliminate contaminating cells and promote astroglia differentiation. This method allows the establishment of primary cell cultures with a degree of purity of at least ∼90% (Braz et al., 2020), which we assessed by the immunostaining of proteins markers specifically enriched in neurons (TUBB3), oligodendroglia (OLIG2), oligodendrocytes (MBP), and astrocytes (GFAP).

#### Foci and Signs of RNA Toxicity

**FIGURE 2:**
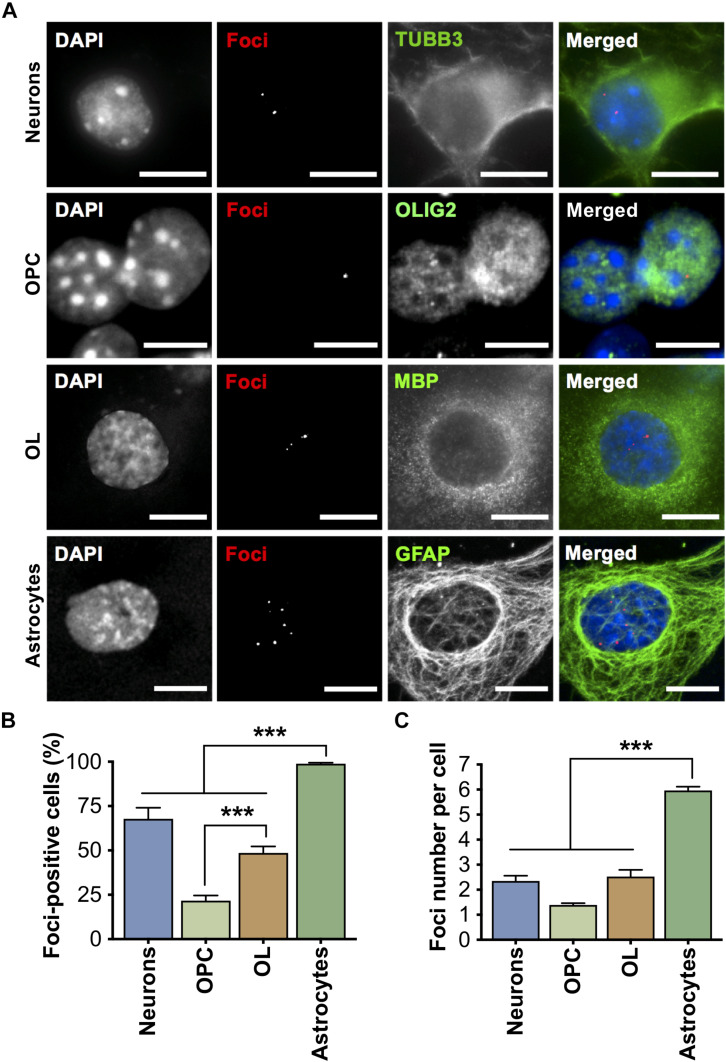
Accumulation of expanded CUG ribonuclear foci in primary DMSXL brain cells. **(A)** Foci accumulation in DMSXL neurons, OPC, OL, and astrocytes was detected by FISH, in combination with immunofluorescence of cell type-specific markers (Neurons, TUBB3; OPC, OLIG2; OL, MBP; Astrocytes, GFAP). Scale bars represent 10 μm. **(B)** Percentage of cells containing at least one focus. Data are average (±SEM). **(C)** Quantification of the number of foci per nucleus. Data are average (±SEM), *n* ≥ 7 cultures per cell type, *n* ≥ 125 cells per cell type (***p < 0.001; one-way ANOVA).

All cell types isolated from DMSXL brains accumulated nuclear CUG RNA foci in variable number (Figure 2A), which demonstrates the expression of expanded *DMPK* transcripts. Primary astrocytes exhibited the most pronounced RNA accumulation, since nearly all cells contained RNA foci (Figure 2B), with a significantly higher average of approximately six foci per nucleus, when compared to other cell types (Figure 2C). Neurons presented foci in around 70% of cells, with a total average of ∼2.4 foci per nucleus. Interestingly, the percentage of oligodendroglia cells (OPC and OL) with foci increased significantly during oligodendrocyte differentiation in culture, from ∼20% in OPC to nearly 50% in OL, with a two-fold increase in the average number of foci per cell (from ∼1.4 in OPC, to 2.5 in OL). MBNL proteins co-localized with nuclear RNA foci in primary DMSXL brain cells, with the exception of MBNL1, which did not show obvious co-localization in DMSXL neurons (Supplementary Figure 1). Interestingly, MBNL1 and MBNL2 showed cytoplasmic localization in primary neurons and astrocytes.

### RNA Sequencing

We performed RNA sequencing to evaluate the effect of expanded CUG repeats on the transcriptome of individual cell types, and gain insight into the affected pathways. In particular, we used this technique to compare the transcriptome of primary neurons, OPC, OL, and astrocytes isolated from DMSXL mice with those collected from WT controls.

#### Expression Analysis

**FIGURE 3:**
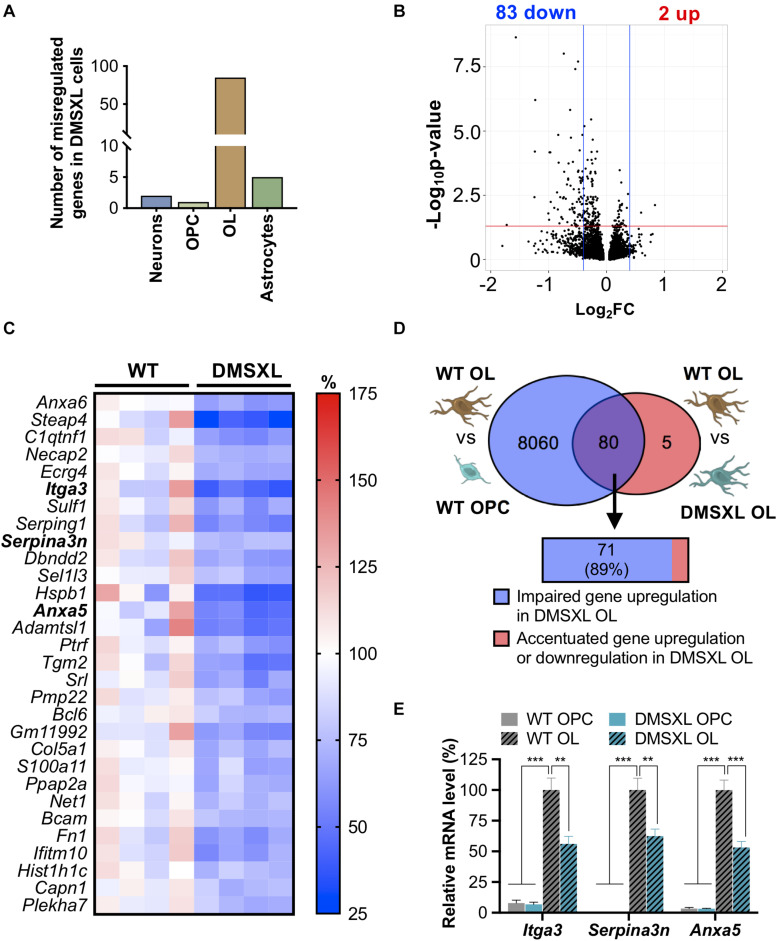
Expression changes in DMSXL brain cells and impaired upregulation of gene expression in oligodendroglia. **(A)** Number of genes differentially expressed in each DMSXL brain cell type. Inclusion criteria: absolute fold change > 1.4; *p* (corrected for multiple comparisons) < 0.05. Total number of gene transcripts identified: neurons, 17,089; OPC, 15,845; OL, 16,070; Astrocytes, 16,878. **(B)** Volcano plot representing the dysregulated transcripts in DMSXL OL, showing that most of the expression defects resulted in gene downregulation, when compared to WT controls. **(C)** Heatmap representing the expression changes of the 30 most significantly affected genes in DMSXL OL, relative to normalized expression in WT OL. Inclusion criteria: absolute fold-change > 1.4, *p* (corrected for multiple comparisons) < 0.001, and base-mean expression value > 50. Genes highlighted in bold were validated by RT-qPCR. **(D)** Overlap of the 85 expression changes in DMSXL OL with the variations that accompany WT oligodendroglia differentiation in culture (representation factor: 2.4, *p* < 1.9E-27). The majority of the overlapping genes failed to upregulate their expression to match the levels of WT OL (Fisher’s exact test, *p* < 0.001). **(E)** RT-qPCR quantification of *Itga3*, *Serpina3n*, and *Anxa5* transcripts in DMSXL OL, confirming lower total expression relative to WT cells after differentiation in culture (**p < 0.01; ***p < 0.001; one-way ANOVA; *n* = 3 OPC; *n* = 6 OL independent cultures per genotype).

We fist studied variations in gene expression. RNA sequencing confirmed the expression of human *DMPK*, *DMWD*, and *SIX5*, the three genes included in the transgene (Seznec et al., 2000), and the downregulation of endogenous *Fbxl7* (Supplementary Table 2). Among all the brain cell types studied, OL showed the highest number of expression changes: the overall transcript levels of 85 genes were significantly modified in DMSXL OL, relative to WT OL. In contrast, DMSXL neurons, OPC and astrocytes displayed a limited number of differentially expressed genes (Figure 3A). The low number of altered transcripts found in DMSXL OPC indicates that the expression differences detected in DMSXL OL emerged during oligodendroglia differentiation, from OPC to OL, and were mostly absent at early precursor cell stages.

We investigated the expression differences in DMSXL OL in further detail, and found that only two events corresponded to RNA upregulation, while all the remaining alterations resulted in the downregulation of steady-state levels of mRNA transcripts (Figures 3B,C). The striking predominance of gene downregulation in DMSXL OL relative to WT OL pointed to the possibility that the affected genes are naturally upregulated during the differentiation and maturation of WT oligodendroglia, but they fail to undergo similar activation in DMSXL cells. To test this hypothesis, we explored the transcriptome shift that occurs during the maturation of OPC into OL in WT cultures. We found 8,140 genes whose total expression varies throughout oligodendroglia differentiation in culture. Remarkably, out of the 85 genes significantly misregulated in DMSXL OL, the vast majority (80/85, 94%) overlapped with this list, presenting differentiation-dependent gene regulation (Figure 3D). In other words, our results suggest that expanded CUG RNA affects primarily the expression of genes that are regulated during oligodendroglia differentiation. Furthermore, and in line with our hypothesis, 71 of those 80 genes (89%) failed to match the upregulation that accompanies the OPC—OL transition in WT cultures, corresponding to a significant overrepresentation of defective gene expression activation in DMSXL OL (Fisher’s test, *p* < 0.001). The remaining eight gene expression variations found in DMSXL OL accentuated the upregulation or downregulation that occurs naturally during the OPC—OL transition in WT cell cultures (Figure 3D). To validate the partial retention of OPC-associated expression profiles in DMSXL OL, we quantified the total transcript levels of three dysregulated genes by RT-qPCR (Figure 3E). The analysis confirmed a pronounced increase in the expression of *Itga3*, *Anxa5* and *Serpina3n* during WT oligodendroglia differentiation, from OPC to OL cell stage. Notwithstanding a modest upregulation during DMSXL oligodendroglia differentiation, the steady-state levels of these three transcripts remained significantly lower in DMSXL OL, relative to WT OL controls.

**FIGURE 4:**
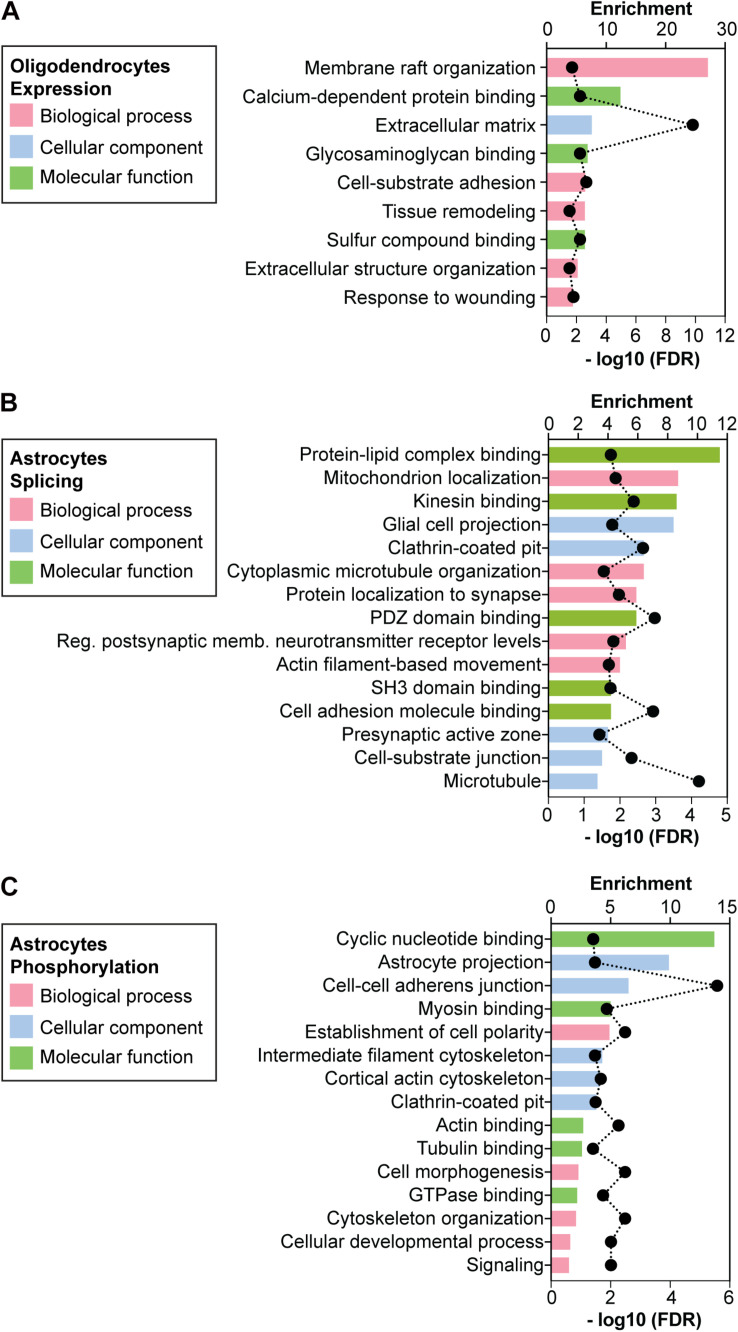
Gene ontology of expression, splicing and phosphorylation changes detected in primary DMSXL cells. Enriched non-redundant GO terms associated with the genes showing **(A)** altered expression levels in primary DMSXL OL, **(B)** variations in alternative splicing in primary DMSXL astrocytes, and **(C)** changes in protein phosphorylation in primary DMSXL astrocytes. The graphs represent the enrichment ratio and the -log10(FDR) of enrichment. A maximum of 5 terms are presented for each ontology (biological process, cellular component and molecular function).

To explore the biological functions associated with the 85 genes differentially expressed in DMSXL OL, we computed the enrichment of biological processes, cellular components and molecular functions by GO analysis. The most highly and significantly enriched GO terms are shown in Figure 4A. Although the GO terms found were diverse, they were frequently related to cell membrane-dependent processes, like cell adhesion, cell morphogenesis, and extracellular matrix (Supplementary Table 3).

In summary, the transcriptome of primary DMSXL brain cells revealed a number of expression changes in oligodendrocytes, which are mainly characterized by the impaired activation of genes that are usually upregulated during oligodendroglia transition, from precursor OPC stages to differentiated OL.

#### Splicing Analysis

**FIGURE 5:**
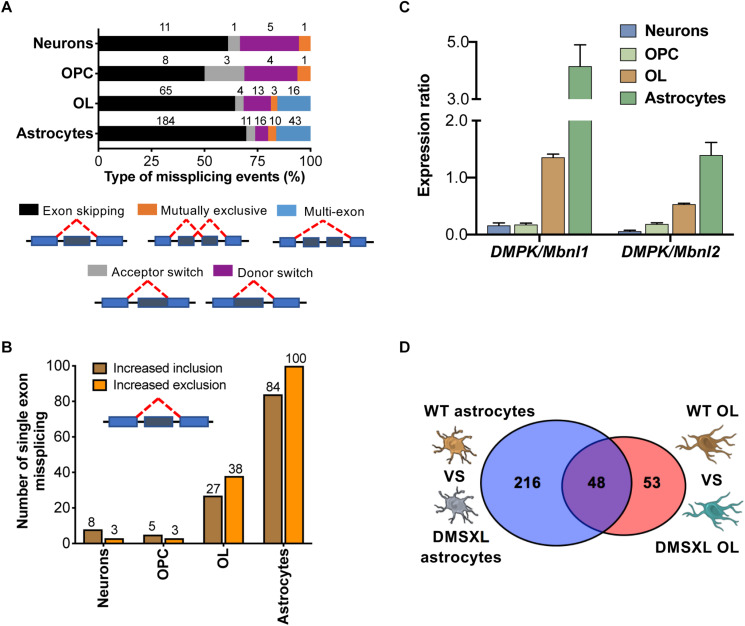
Overview of splicing changes detected in DMSXL brain cells. **(A)** Distribution of all types of alternative splicing abnormalities studied among the different brain cell types. Percentage of events in each category. Absolute numbers are shown. **(B)** Number of cassette exons showing increased inclusion (positive ΔPSI) or exclusion/skipping (negative ΔPSI) in primary DMSXL cells, compared to WT controls. **(C)** Expression rations for human *DMPK* vs. total mouse *Mbnl1* and *Mbnl2* transcripts across brain cell types. **(D)** Venn Diagram showing overlapping splicing events dysregulated in DMSXL astrocytes and or DMSXL OL (representation factor: 24.0; *p* = 1.48E-11).

We then interrogated the RNA sequencing data to investigate the alternative splicing profile of DMSXL brain cells. We studied five different types of splicing events: single and multiple exon skipping, mutually exclusive exons, and acceptor/donor site switch (Figure 5A and Table 1). Among the four types of brain cells studied, astrocytes displayed the highest number of splicing changes (264), followed by OL (101), neurons (18), and OPC (16). In all cell types, the most prevalent modifications comprised single exons (Figure 5B). In DMSXL astrocytes, 184 cassette exons showed increased inclusion/exclusion, relative to WT controls. In contrast, primary DMSXL neurons exhibited only 11 significant variations in the splicing of single exons. Interestingly, and similar to the variations in gene expression, the number of cassette exons differentially spliced between DMSXL and WT cells increased from eight in OPC to 65 in OL primary cultures. In an attempt to gain insight into the factors contributing to the more severe spliceopathy of DMSXL astrocytes and OL, we quantified the levels of human *DMPK* transcripts, relative to mouse *Mbnl1* and *Mbnl2*, as previously described (Otero et al., 2021). DMSXL astrocytes displayed the highest *DMPK/Mbnl1* and *DMPK/Mbnl2* ratios, followed by DMSXL oligodendrocytes (Figure 5C).

**TABLE 1:** Examples of splicing changes found in DMSXL astrocytes for each of the five categories of events studied.

Gene	Type of event	Exon number^a^	Exon size (bp)	Δ PSI^b^	*p*-value^c^	Δ PSI DM1 FC^d^	Δ PSI DM1 FC^e^
*Palm*	Single exon skipping	8	132	−33	3.03E-04	−31	−38
*Capzb*	Single exon skipping	11	113	−34	<1E-10	−6 (n.s.)	−10
*Inf2*	Single exon skipping	22	57	−52	8.84E-06	−7 (n.s.)	N/D
*Numa1*	Single exon skipping	16	42	−34	5.04E-07	−21	+ 26
*Itga6*	Multi-exon skipping	26/27	373/130	−38	3.01E-09	−27	−33
*Dnm2*	Mutually exclusive	10/11	139/139	39	1.09E-03	N/D	N/D
*Magi1*	Acceptor switch	22	204	24	3.28E-03	N/D	N/D
*Slc39a11*	Donor switch	6	145	25	5.77E-10	N/D	N/D

*^a^Exons were numbered according to the FasterDB web interface (http://fasterdb.ens-lyon.fr/).*

*^b^ΔPSI, difference between the percentage of exon inclusion in DMSXL and WT cells.*

*^c^Fisher’s test, corrected for multiple comparisons.*

*^d^ΔPSI reported in DM1 human frontal cortex, relative to non-affected individuals (Otero et al., 2021).*

*^e^ΔPSI reported in DM1 human frontal cortex, relative to non-affected individuals (Goodwin et al., 2015). n.s., not significant; N/D, not determined.*

We explored potential overlaps in splicing dysregulation between brain cell types. Among the five categories studied (Figure 5A), we found a significant overlap of 48 events dysregulated in primary DMSXL astrocytes and DMSXL OL (*p* = 1.48E-11), but none of them was shared with DMSXL neurons or DMSXL OPC (Figure 5D). In addition to *Mbnl1* exon 7 and *Mbnl2* exon 9, other overlaps between DMSXL astrocytes and DMSXL OL included *Capzb* exon 11, *Dmd* exon 81, *Inf2* exon 22, and *Numa1* exon 16. Interestingly, some missplicing events were specifically detected in individual DMSXL cell types, indicating differences at the cell type level (e.g., *Osbp* exon 10 was exclusively misspliced in DMSXL neurons, *Nek3* exon 11 in DMSXL OPC, *Ctnnd1* exon 5 in DMSXL OL, *Ktn1* exon 41 in DMSXL astrocytes).

**FIGURE 6:**
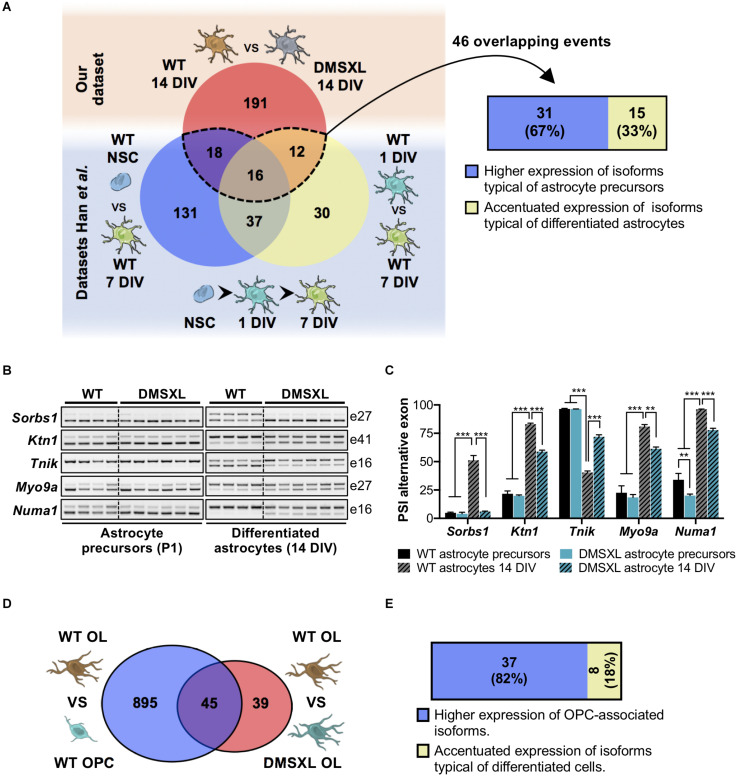
Analysis of DMSXL splicing profiles during glia cell differentiation. **(A)** Significant overlap of DMSXL astrocytes splicing defects with changes occurring during WT astroglia differentiation (representation factor: 23.4, *p* < 1.7E-49). A significant fraction of overlapping splicing changes resulted in the upregulation of RNA isoforms typical of less differentiated astrocytes (Fisher’s exact test, *p* = 0.023). **(B)** RT-PCR analysis of alternative cassette exons with impaired or partial splicing switch during astrocyte differentiation, from isolated precursors (P1) to astrocytes differentiated in culture (14 DIV). Alternative exon numbers are indicated on the right. **(C)** PSI of alternative exons in astrocyte precursor and astrocytes differentiated in culture (14 DIV), collected from DMSXL and WT mice (**p < 0.01; ***p < 0.001; one-way ANOVA; *n* = 4 WT, *n* = 6 DMSXL, independent cultures). **(D)** Overlap of splicing variations concomitant to WT oligodendroglia transition, with the dysregulated splicing events in DMSXL OL (representation factor: 16.5, *p* < 1.4E-44). **(E)** Significant overrepresentation of splicing changes that increase the expression of OPC-associated RNA isoforms in DMSXL OL (Fisher’s exact test, *p* = 0.003). Drawings of brain cells from Servier Medical Art, licensed under a Creative Commons Attribution 3.0 Unported License (CC BY 3.0).

Given the pronounced splicing dysregulation in DMSXL astrocytes, we focused our subsequent analyses on this glial cell type. We calculated the enriched GO terms associated with the misspliced transcripts in DMSXL astrocytes (Figure 4B and Supplementary Table 4), and found terms relevant for glial cell function, related to membrane cell projections, cytoskeleton, synapse and cell adhesion. We then asked whether the splicing profiles of DMSXL astrocytes provided evidence of impaired splicing transitions during differentiation, similar to the expression changes in DMSXL oligodendroglia. To address this question, we used previously published datasets, which included transcriptomic data from mouse NSC, immature astrocytes (collected at 1 DIV) and differentiated astrocytes (collected at 7 DIV) (Han et al., 2014). We generated a list of 244 physiological splicing variations associated with astroglia differentiation and maturation involving single exons, multiple exons or mutually exclusive exons (Figure 6A): 202 changes occurred during the NSC – 7 DIV transition and an additional 95 took place during the final 1 DIV – 7 DIV maturation (with an overlap of 53 splicing events between both). Then, we crossed this list with all single, multiple or mutually exclusive exons differentially spliced in DMSXL astrocytes. We found a significant overlap of 46 splicing events, which not only accompanied astroglia differentiation in culture, but were also significantly affected in DMSXL astrocytes. Among differentiation-dependent alternative exons affected in DMSXL astrocytes, the majority increased the expression of isoforms typical of immature astroglia (Figure 6A): incomplete splicing transitions in DMSXL astrocytes were significantly over-represented, relative to splicing variations that increased the expression of RNA isoforms predominant in differentiated astrocytes (Fisher’s exact test, *p* = 0.0232).

To further validate this observation, we performed a new series of experiments. We purified astrocyte precursors from newborn mouse cortex, using microbeads conjugated to a pan-astrocyte marker (ACSA-2), extracting RNA immediately upon isolation (astrocyte precursors) and after 14 DIV (differentiated astrocytes). RT-PCR analysis of selected alternative exons in DMSXL astrocytes at 14 DIV confirmed the expression of splicing isoforms that are typically more abundant in precursor cell stages (P1) (Figure 6B and Supplementary Figure 2). In particular, the immature splicing profile of *Sorbs1* exon 27 was fully retained during DMSXL astrocyte differentiation, matching the profile of poorly differentiated astrocytes collected at P1. Although the splicing of *Ktn1* exon 41, *Tnik* exon 16, *Myo9a* exon 27, and *Numa1* exon 16 changed during the differentiation of DMSXL astrocytes, the PSI of alternative exons did not reach the values found in differentiated WT astrocytes (Figure 6C).

Similarly, DMSXL oligodendroglia also exhibited splicing differences suggestive of impaired differentiation: out of the 84 splicing alterations (involving single, multiple or mutually exclusive exons) detected in DMSXL OL, 45 (54%) affected alternative exons regulated during cell differentiation (Figure 6D). Among those, the majority (37 out of 45) increased the relative abundance of RNA isoforms typically expressed in OPC. Only eight splicing modifications in DMSXL OL resulted in the abnormally high expression of isoforms characteristic of differentiated OL (Figure 6E).

In summary, primary DMSXL astrocytes and oligodendrocytes exhibit missplicing events that affect a significant proportion of differentiation-regulated alternative exons. In most cases those events increase the expression of splicing isoforms naturally expressed in undifferentiated or precursor cells stages.

#### Exon Ontology and Functional Implications of Splicing Changes

**FIGURE 7:**
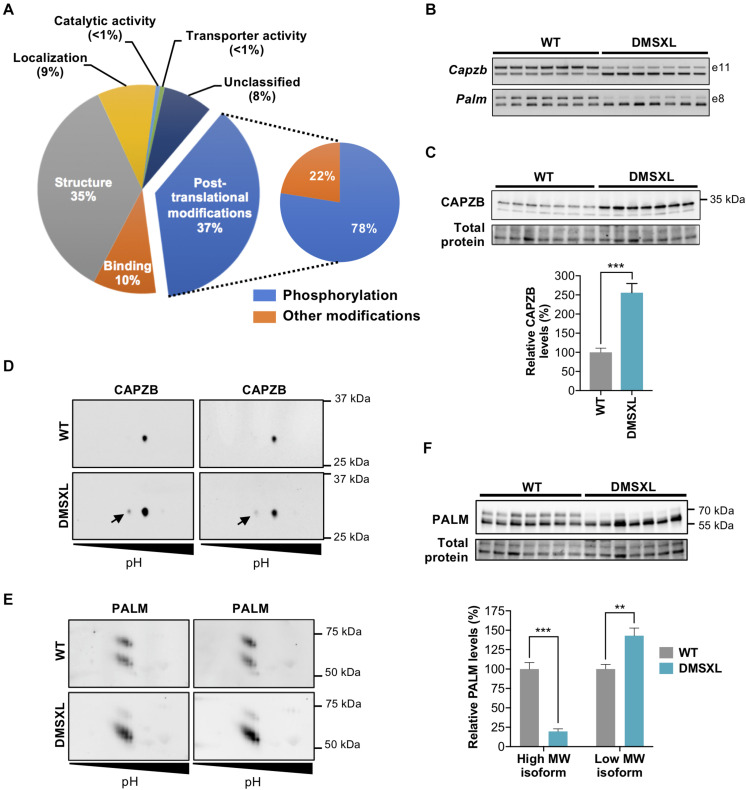
Exon ontology analysis and phosphoproteomic analysis of DMSXL astrocytes. **(A)** Exon ontology analysis shows the impact of splicing changes on several features of proteins of primary DMSXL astrocytes. **(B)** Representative RT-PCR analysis of alternative splicing of *Capzb* exon 11 and *Palm* exon 8 in primary mouse astrocytes, showing pronounced missplicing in DMSXL cells (*n* = 7 independent cultures, per genotype). **(C)** Western blot analysis of CAPZB, showing upregulation of C-terminal protein isoform in DMSXL astrocytes (***p < 0.001; Student *t*-test; *n* = 7 per group). **(D)** 2D-WB phosphorylation analysis of CAPZB, showing the expression of a phosphorylated form (arrow) in DMSXL astrocytes (*n* = 2 independent cultures, per genotype). **(E)** Two-dimension western blot of PALM showing abnormal protein isoform between genotypes, revealing low levels of a high molecular weight isoform in DMSXL astrocytes (*n* = 2 independent cultures, per genotype). **(F)** Western blot quantification showing the changes in the relative abundance of PALM protein isoforms: downregulation of the high molecular weight isoform, and upregulation of low molecular weight isoform (**p < 0.01; ***p < 0.001; Mann Whitney *U* test; *n* = 7 independent cultures per genotype).

The high number of exons differentially spliced in DMSXL astrocytes prompted us to perform an exon ontology analysis (Tranchevent et al., 2017) to gain insight into the biological role the protein domains encoded, and shed light on the functional consequences of the splicing alterations. We identified several protein features that may be selectively impacted by missplicing, including post-transcriptional modifications (37%), protein structure (35%), binding (10%), or intracellular localization (9%) (Figure 7A). Among post-translational modifications, the majority affected protein phosphorylation.

### Analysis of DMSXL Astrocyte Phosphoproteome

To investigate changes in the phosphoproteome of DMSXL astrocytes, we performed a global phosphoproteomics approach (Supplementary File 1). We identified 288 proteins in primary DMSXL astrocytes showing significant phosphorylation changes in at least one phosphosite, relative to WT controls, corroborating the impact of the repeat expansion on the phosphorylation of target proteins. Interestingly, amidst the total of 345 phosphosites perturbed in DMSXL astrocytes, we found a significant over-representation of increased phosphorylation (279 events), vs. decreased phosphorylation (66 events; *p* < 0.001, Fisher’s exact test). We used kinase enrichment analysis (Lachmann and Ma’ayan, 2009) to infer upstream kinases whose putative substrates were overrepresented in hyperphosphorylated proteins. We found a significant enrichment for members of key signaling pathways (Table 2).

**TABLE 2:** Top candidate kinases with overpresented hyperphoshprylated substrates in DMSXL astrocytes.

Kinase	Signaling pathway	Overlap^a^	*p*-value^b^	Molecular abnormality
GSK3B	AKT, AMPK	45/527	1.59E-24	No change
RPS6KA	ERK, MAPK	34/332	4.22E-21	S715 hyperphosphorylation
PRKCB	Protein kinase C	23/250	3.15E-13	No change
MAPK14	MAPK	26/396	1.31E-11	Y182 hyperphosphorylation
PRKACA	cAMP	25/393	6.49E-11	No change
PRKCA	Protein kinase C	26/442	1.19E-10	No change
MAPK9	MAPK	14/131	3.66E-09	No change
AKT1	AKT	17/210	3.66E-09	No change
MAPK8	MAPK	17/225	9.60E-09	No change
MAPK1	MAPK	20/326	1.08E-08	No change

*^a^Fraction of putative kinase substrates hyperphosphorylated in DMSXL astrocytes.*

*^b^Fisher’s test, corrected for multiple comparisons.*

Among those 288 proteins abnormally phosphorylated in DMSXL astrocytes, 27 were encoded by transcripts misspliced in this cell type, revealing a significant overlap (*p* < 1.01E-21). Enriched GO terms associated with the proteins differentially phosphorylated in DMSXL astrocytes were related to cyclic nucleotide binding and signaling, cytoskeleton protein binding, cell morphology and adhesion (Figure 4C and Supplementary Table 5).

We selected two candidate proteins for validation: CAPZB and PALM. We first confirmed the marked missplicing of *Capzb* and *Palm* transcripts in DMSXL astrocytes (Figure 7B and Supplementary Figure 3A). The exclusion of *Capzb* exon 11 shifts the translation stop codon further downstream, changing the C-terminal sequence of the protein (Schafer et al., 1994). Using a C-terminal specific antibody, we confirmed a ∼three-fold isoform protein upregulation in DMSXL astrocytes (Figure 7C and Supplementary Figure 3B). We then sought to validate protein phosphorylation changes by two-dimension western blot (2D-WB), and detected a low abundant phosphorylated CAPZB protein isoform specifically in DMSXL astrocytes, which was not detected in WT cells (Figure 7D and Supplementary Figure 3C). The 2D-WB analysis of PALM did not reveal clear differences in the isoelectric point between the two genotypes, which would be indicative of altered protein phosphorylation. Instead, we found an intriguing difference in the relative abundance of two isoforms presenting different molecular weights (Figure 7E and Supplementary Figure 3D), which we confirmed by quantitative western blot analysis with an antibody directed against the full protein (Figure 7F and Supplementary Figure 3E).

Our data demonstrate the impact of the spliceopathy of DMSXL astrocytes on multiple features of downstream proteins.

### Analysis of Protein Localization in DMSXL Astrocytes

**FIGURE 8:**
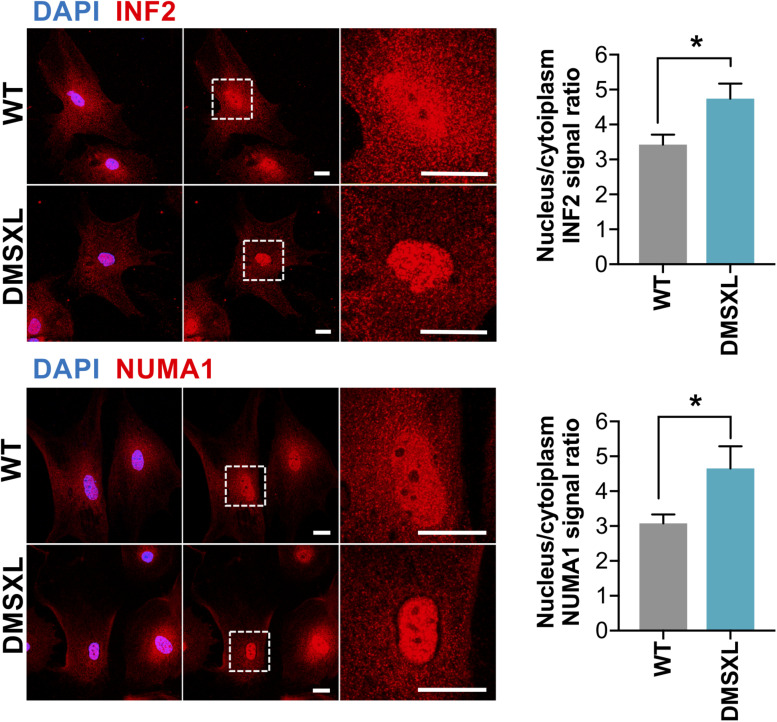
Altered protein localization in DMSXL astrocytes. Representative confocal images and quantification of the distribution INF2 and NUMA1 proteins between the nucleus and cytoplasm in primary DMSXL and WT astrocytes. Protein signal was lower in the perinuclear cytoplasm of DMSXL astrocytes compared to WT controls cells. Scale bars represent 20 μm (*p < 0.05; Student *t*-test *U* test; WT, *n* = 16 cells, DMSXL, *n* = 15 cells; 2 independent cultures per genotype).

To functionally validate the impact of alternative splicing dysregulation on the intracellular distribution of astrocyte proteins, we selected INF2 and NUMA1, which showed pronounced RNA missplicing of alternative exons predicted to regulate intracellular protein localization (Figure 6B and Supplementary Figure 4). Immunofluorescence confirmed modified subcellular distribution of INF2 and NUMA1 in cultured DMSXL astrocytes, when compared to WT control cells (Figure 8). Cytoplasmic localization was decreased in DMSXL cells (particularly in the perinuclear region), leading to a significant increase in the nuclear/cytoplasmic signal ratio of both proteins. These results support the association between RNA missplicing in DMSXL astrocytes and some functional features of encoded proteins, including their intracellular localization, and in particular their nuclear/cytoplasmic distribution. The absence of overt changes in the distribution of SORBS1 and CLASP1, in DMSXL astrocytes (Supplementary Figure 5), reveals that protein mislocalization affects a fraction of proteins encoded by misspliced transcripts.

## Discussion

The role of glial cells in DM1 brain dysfunction has been eclipsed by the focus of research on the neuron-specific root causes of neuropsychological manifestations (Charizanis et al., 2012; Hernández-Hernández et al., 2013; Wang et al., 2017, 2018; Lee et al., 2019). The need to characterize and distinguish disease-related events taking place in individual brain cell types prompted us to compare the transcriptome of primary neurons, astrocytes and oligodendrocytes derived from a relevant disease mouse model. The importance of our study is the finding that CUG RNA expression and accumulation can induce different degrees of gene expression and splicing changes in neuronal and non-neuronal primary brain cells, affecting the metabolism of downstream proteins in different ways.

Prior to the molecular analysis, we verified that all major brain cell types expressed and accumulated expanded mutant *DMPK* transcripts, through the analysis of ribonuclear foci. Although we could detect foci in all the cell types, we observed a significantly higher number in astrocytes. RNA sequencing of DMSXL primary cells revealed that astrocytes, OPC and neurons were largely unaffected by changes in gene expression, pointing to the possibility that the mechanisms of transcriptional regulation in these cells are not disturbed by expanded CUG repeats. In contrast, a substantial number of changes was detected in DMSXL OL. Our top downregulated gene in DMSXL OL (*Anxa6*) has been recently found downregulated in DM1 frontal cortex, and proposed as a candidate CSF marker to study disease progression (Otero et al., 2021). An additional six genes downregulated in DMSXL OL were also described to be differentially expressed in DM1 frontal cortex. We currently do not know why this cell type, with lower abundance of RNA foci relative to astrocytes, shows more dramatic variations in gene expression in DMSXL cell cultures. One possibility is that the regulation of gene expression in cultured DMSXL OL is more susceptible to the accumulation of RNA foci. Interestingly, the expression the mouse *Dmpk* gene shows a two-fold increase from OPC to newly formed OL (Zhang et al., 2014), while the *DMPK/Dmpk* expression ratio is also 50% higher in DMSXL OL. Variations in the expression of the expanded *DMPK* transgene, relative to MBNL, CELF, and other RNA-binding proteins may account for the distinct transcriptome responses to the DM1 expansion, and require further investigation.

When looking particularly at alternative splicing, DMSXL astrocytes showed the highest number of modifications relative to WT cells, which included variations in single and multiple exon skipping, mutually exclusive exons, acceptor and donor site switches. The more severe splicing dysregulation in primary DMSXL astrocytes may be a consequence of the marked accumulation of RNA foci, which co-localize with MBNL proteins, likely impairing their activity. Although protein levels cannot be directly inferred from transcript quantification, the highest *DMPK/Mbnl1* and *DMPK/Mbnl2* ratios in DMSXL astrocytes are in line with this view: DMSXL astrocytes may therefore provide conditions conducive to MBNL protein sequestration and RNA missplicing. The missplicing events shared between DMSXL astrocytes and oligodendrocytes suggest similar mechanisms of splicing regulation across glial cell types. In contrast, cell type-specific splicing defects may affect transcripts that are differentially expressed or that exhibit different susceptibilities to the varying accumulation of toxic CUG RNA.

The transcriptome signature of primary DMSXL OL and astrocytes provided molecular evidence of impaired glial cell differentiation in response to the expanded *DMPK* transcripts. In support of this view, DMSXL OL failed to fully upregulate genes that are naturally activated during oligodendroglia differentiation. Furthermore, both DMSXL OL and DMSXL astrocytes displayed incomplete differentiation-dependent splicing switches, characterized by the high expression of splicing isoforms typically found in precursor or undifferentiated cells, as previously described in the skeletal muscle, heart and brain of DM1 mouse models expressing expanded CTG repeats (Lin et al., 2006; Kalsotra et al., 2008; Hernández-Hernández et al., 2013). This general feature of DM1 splicing signatures can be explained, at least partially, by the key role of MBNL and CELF proteins in the molecular programs of cell differentiation (Ladd et al., 2001; Han et al., 2013). However, the impact on the differentiation and function of brain cells demands further investigation, which will be warranted in our future studies.

Exon ontology uncovered different protein features affected by the splicing defects found in DMSXL astrocytes, such as post-translational modifications and intracellular localization. The comparison between the phosphoproteome and the transcriptome of DMSXL astrocytes revealed that among the 288 proteins differentially phosphorylated in this cell type, a fraction (27 proteins) was encoded by misspliced transcripts, but the majority did not show signs of splicing alterations in our RNA sequencing analysis. These results suggest that phosphorylation changes are not necessarily a direct consequence of the missplicing of the corresponding transcript. Instead, they may be caused by the dysregulation of signaling kinases, which will affect their downstream substrates. We found increased phosphorylation of RPS6KA3 and MAPK14 in DMSXL astrocytes by mass spectrometry, which has been shown to induce kinase activity (Romeo et al., 2012; Asih et al., 2020). The possible contribution of RPS6KA3 and MAPK14 to global changes in the phosphoproteome requires further studies. It is noteworthy that proteins previously described to be hyperphosphorylated in DM1 were either not detected in DMSXL astrocytes by mass-spectrometry analysis (e.g., CELF1, CELF2, and MAPT), or did not show signs of altered phosphosites (e.g., GSK3B).

GO analyses were performed to explore the cellular and functional involvement of the affected genes and their possible contribution to DM1 brain pathology. Despite some variability between cell types, GO revealed that the proteins and/or transcripts perturbed in DMSXL glial cells were related to the cytoskeleton, cell membrane and cell adhesion. During differentiation, cells generate and undergo mechanical forces that shape their morphology, tissues and organs, but that can also regulate genetic programs. Cell adhesion, in particular, is critical for the mechanotransduction of external stimuli into internal signaling cascades, which subsequently drive the complex changes in membrane and cytoskeleton dynamics required to achieve the highly specialized morphology and intricate interactions with neighboring cells (Weigel et al., 2020). Among the target transcripts and proteins affected in DMSXL astrocytes, CAPZB and PALM regulate cell morphology and differentiation, through their interaction with the actin-cytoskeleton and sites of plasma membrane activity (e.g., filopodia), respectively (Arstikaitis et al., 2008; Mukherjee et al., 2016). Both proteins show alternative splicing and phosphorylation-dependent activity (Kutzleb et al., 1998; Solís and Russell, 2019). Extensive prenylation and palmitoylation have been reported in PALM, contributing to the complex electrophoretic profiles (Kutzleb et al., 1998). It is possible that *Palm* missplicing in DMSXL astrocytes impacts a variety of post-translational modifications, explaining the changes in the relative abundance of the two protein isoforms detected. INF2 is an endoplasmic and nuclear envelope membrane-associated protein, which regulates perinuclear actin assembly (Shao et al., 2015). NUMA1 is a structural component of the nucleus with microtubule organizing activity (Nachury et al., 2001; Wiese et al., 2001). The involvement of nuclear envelope transmembrane proteins and the nuclear-cytoskeleton has been previously suggested in DM1 (Hintze et al., 2018). In the future, it would be interesting to determine if the nuclear accumulation of INF2 and NUMA1, as well as the splicing and protein defects of CAPZB and PALM, in DMSXL cells affect the organization and dynamics of the cytoskeleton, and how they correlate to clinical features of DM1.

Different lines of evidence indicate that the abnormalities found in DMSXL cells result from the expression of expanded *DMPK* transcripts, rather than from other human genes present in the transgene, or from integration site effects. First, control DM20 mice expressing the three genes of the human *DM1*, including a short CTG repeat, do not display overt phenotypes or significant molecular changes (Seznec et al., 2001; Huguet et al., 2012; Hernández-Hernández et al., 2013; Sicot et al., 2017). Second, in line with the hypothesis of CUG RNA toxicity, molecular abnormalities were more pronounced in DMSXL astrocytes, which express the highest levels of *DMPK* transcripts, relative to other brain cell types. Third, there is significant overlapping between splicing changes in DMSXL mouse brain cells and those reported in human DM1 frontal cortex (Goodwin et al., 2015; Otero et al., 2021; Supplementary File 2). Fourth, *Fbxl7* knock-out mice have not shown overt neurological phenotypes^5^.

Most neurological diseases can no longer be viewed solely as neuronopathies. Despite some inherent differences between cell culture and *in vivo* systems, our findings demonstrate that astrocytes and OL are determinant players in DM1 brain dysfunction, providing useful experimental systems in which to decipher the mechanisms and intermediates of CUG RNA toxicity in brain cells. Our results do not exclude, however, the implication of neurons in DM1 brain disease. We collected each cell type at stages of development that were selected to maximize cell yield and viability in culture. It has been shown that *DMPK* transgene expression and foci accumulation vary throughout DMSXL mouse embryonic development and aging (Michel et al., 2015). Therefore, it is possible that DMSXL neurons collected at other differentiation stages may show more pronounced alterations in gene expression and alternative splicing. Further studies are warranted to determine the extent to which the impact of the DM1 CTG trinucleotide repeat expansion on brain cells varies with developmental stage, cellular differentiation and aging.

We have leveraged multi-omics approaches in a mouse model of DM1, to address how expanded CUG RNA transcripts impact gene expression, alternative splicing and downstream protein features in different brain cell types. We gathered molecular evidence of the dysregulation of expression and splicing differentiation programs in glial cells. Our results lay important tools and groundwork to further decipher the cellular mechanisms behind DM1 brain disease.

## Data Availability Statement

The datasets generated for this study by RNA sequencing can be found in the Gene Expression Omnibus repository, https://www.ncbi.nlm.nih.gov/geo/query/acc.cgi?&acc=GSE162093. Phosphoproteomics datasets generated for this study can be found in the PRIDE repository: http://www.ebi.ac.uk/pride/archive/projects/PXD025011.

## Ethics Statement

This project was conducted according to the ARRIVE guidelines (Animal Research: Reporting *in vivo* Experiments), with the authorization for animal experimentation number 75 003 in the animal facility, with the approval number B 91 228 107, both delivered by Prefecture de police and the French veterinary department.

## Author Contributions

AG-B, ICG, CFB, DA, GG, and MG-P: conceptualization. DD and SB: methodology. AG-B and LL: validation. AG-B, HP, IG, J-BC, CFB, and MG-P: formal analysis. AG-B, LL, DD, SB, PM, CP, AH-L, and CC: investigation. ICG, CB, DA, GG, and MG-P: resources. AG-B, ICG, HP, and CFB: data curation. AG-B and MG-P: writing—original draft. AG-B, ICG, CFB, DA, GG, and MG-P: writing—review and editing. AG-B, LL, and MG-P: visualization. ICG, CFB, DA, GG, and MG-P: supervision. MG-P: project administration. CFB, DA, GG, and MG-P: funding acquisition. All authors contributed to the article and approved the submitted version.

## Conflict of Interest

AG-B was a former employee of BioMarin Pharmaceutical Inc., and holds shares of the company. The remaining authors declare that the research was conducted in the absence of any commercial or financial relationships that could be construed as a potential conflict of interest.

## Footnotes

1 http://icy.bioimageanalysis.org

2 http://nemates.org/MA/progs/representation.stats.html

3 http://www.webgestalt.org/

4 https://maayanlab.cloud/Enrichr

5 https://www.mousephenotype.org
