# Supplementary material for: Integrative Cell Type-Specific Multi-Omics Approaches Reveal Impaired Programs of Glial Cell Differentiation in Mouse Culture Models of DM1
Source: Front Cell Neurosci. 2021 May 5;15:662035. doi: 10.3389/fncel.2021.662035 (PMC8136287; doi:10.3389/fncel.2021.662035)
Supplement: Supplementary file 1 [file Presentation_1.pdf]

Supplementary Material

Integrative Cell Type-Specific Multi-Omics Approaches Reveal Impaired Programs of Glial Cell Differentiation in Mouse Culture Models of DM1

Anchel González-Barriga[1], Louison Lallemant[1,†], Diana M. Dincã[1,†], Sandra O. Braz[1,2,†], Hélène Polvèche[3,4], Paul Magneron[1], Cédric Pionneau[5], Aline Huguet-Lachon[1], Jean-Baptiste Claude[3], Cerina Chhuon[6], Ida Chiara Guerrera[6], Cyril F. Bourgeois[3], Didier Auboeuf[3], Geneviève Gourdon[1], Mário Gomes-Pereira[1,*]

[1] Sorbonne Université, Inserm, Institut de Myologie, Centre de Recherche en Myologie, F-75013 Paris, France.

[2] Inserm UMR1163, Institut Imagine, Université Paris Cite, F-75015 Paris, France.

[3] Laboratory of Biology and Modeling of the Cell, Université de Lyon, ENS de Lyon, Université Claude Bernard, CNRS UMR 5239, Inserm U1210, F-69007 Lyon, France.

[4] Inserm/UEVE UMR861, Université Paris Saclay I-STEM, F-91110 Corbeil-Essonnes, France.

[5] Sorbonne Université, Inserm, UMS PASS, Plateforme Post-génomique de la Pitié Salpêtrière (P3S), F-75013, Paris, France.

[6] Proteomics Platform Necker, Université de Paris - Structure Fédérative de Recherche Necker, Inserm US24/CNRS UMS3633, F-75015 Paris, France.

[†] These authors have contributed equally to this work.

[*]**Correspondence:**

Mário Gomes-Pereira: mario.pereira@inserm.fr

1 Supplementary Data

Supplementary File 1. Phosphoproteomics data analysis of DMSXL astrocytes.

List of phosphosites deregulated in DMSXL astrocytes, including gene names, protein and unique ID, with the adjusted *p* value, log2 fold change in DMSXL cells compared to WT controls (Log2FC) and phosphorylation position of the corresponding amino acid in the peptide sequence (sequence window). Phosphosites uniquely detected in one condition were assigned an arbitrary *p* value of 2.4E-5 and Log2FC of +3 (if hyperphosphorylated), or -3 (if hypophosphorylated). Phosphoproteomics datasets generated for this study can also be found in the PRIDE repository: http://www.ebi.ac.uk/pride/archive/projects/PXD025011.

Supplementary File 2. Comparison of splicing dysregulation in DMSXL mouse brain cells and human DM1 frontal cortex.

Orthologous exons dysregulated in DMSXL mouse cells (astrocytes and oligodendrocytes) and in human frontal cortex (Goodwin et al. 2015; Otero et al. 2021). Exons are numbered according to the FasterDB web interface (http://fasterdb.ens-lyon.fr/faster/home.pl). *Exons dysregulated in opposite directions between DMSXL mouse cells and DM1 frontal cortex (inclusion/exclusion).

2 Supplementary Figures and Tables

2.1 Supplementary Tables

Supplementary Table 1. Oligonucleotide primer sequences.

Genes symbols	Aim	Alternative exon[a]	Exon Size (bp)	Oligonucleotide primer sequences (5'>3')
Anxa5	Expression analysis	N/A	N/A	ACTCCTGACCGACAGCATCAT<br>GCATCAGCCCTGCCATCAAAT
Itga3	Expression analysis	N/A	N/A	ACGACTGTGAACGGATGGAC<br>GGGCACAGACCAGGACTCTA
Polr2a	Expression analysis	N/A	N/A	GGCTGTGCGGAAGGCTCTG<br>TGTCCTGGCGGTTGACCC
Serpina3n	Expression analysis	N/A	N/A	AGGAAACAGACCCAGGGGAT<br>GGCACCTTCCATTTGGCTTTA
Capzb	Splicing analysis	11	113	GCACGCTGAATGAGATCTACTTTG<br>CCGGTTAGCGTGAAGCAGAG
Inf2	Splicing analysis	22	57	CTGAAGATACCCCGGATGCC<br>CCGACGAGAGCACTCACTTG
Ktn1	Splicing analysis	41	84	TGAGAAGAGAAAGAGAGCATTTG<br>GTGTGTTTCATTTAACTGTGTCTTG
Myo9a	Splicing analysis	27	213	GCCCACAAACAAGATGAATCAGC<br>CAGAAAATGGATGAGCAGGTGTC
Numa1	Splicing analysis	16	42	GACCCACTTGGCTGAAATGC<br>GTCAGCTTCTTACTTAGTTCTTCC
Palm	Splicing analysis	8	132	GGAGCAAAAGTCAGAAACCTTGG<br>TTGTGAATGAGTTCGTCCACCTC
Sorbs1	Splicing analysis	27	168	CCAGCTGATTACTTGGAGTCCACAGAAG<br>GTTCACCTTCATACCAGTTCTGGTCAATC
Tnik1	Splicing analysis	16	87	GGCACTACGAAGAACAGATGCG<br>GCTGAACCCCACTAATGCTGAAG

[a]Alternative exons are numbered according to the FasterDB web interface (http://fasterdb.ens-lyon.fr/faster/home.pl).

Supplementary Table 2. Expression levels of human genes in the transgene, relative to mouse endogenous orthologues. Mean number of *Fbxl7* transcripts per million is shown. Data are mean (±SEM). N/D, not detected.

	Neurons		OPC		OL		Astrocytes	
	WT	DMSXL	WT	DMSXL	WT	DMSXL	WT	DMSXL
DMPK/Dmpk		0.53±0.16	0	0.40±0.03	0	0.63±0.02	0	2.21±0.59
DMWD/Dmwd	0	0.03±0.002	0	0.08±0.02	0	0.17±0.01	0	1.08±0.10
SIX5/Six5	0	16.06±4.78	0	2.69±0.16	0	3.60±0.33	0	5.97±1.24
Fbxl7	0.18±0.02	N/D	0.67±0.08	0.04±0.02	0.70±0.09	0.05±0.02	4.59±0.35	0.50±0.07

Supplementary Table 3. Gene ontology analysis of genes differentially expressed in DMSXL OL. The most highly enriched, non-redundant, significant terms in each ontology are listed. A maximum of five terms are shown per ontology (biological process, cellular component and molecular function), in decreasing order of enrichment ratio.

GO code	Term	Enrichment ratio	FDR
**Biological process**			
GO:0031579	Membrane draft organization	27.1	2.0E-02
GO:0031589	Cell-substrate adhesion	6.5	2.2E-03
GO:0048771	Tissue remodeling	6.4	2.9E-02
GO:0043062	Extracellular structure organization	5.2	2.9E-02
GO:0045785	Response to wounding	4.4	1.6E-02
**Cellular component**			
GO:0031012	Extracellular matrix	7.6	1.5E-10
**Molecular function**			
GO:0048306	Calcium-dependent protein binding	12.4	5.8E-03
GO:0005539	Glycosaminoglycan binding	6.9	5.8E-03
GO:1901681	Sulfur compound binding	6.4	5.8E-03

Supplementary Table 4. Gene ontology analysis of genes differentially spliced in DMSXL astrocytes. The most highly enriched, non-redundant, significant terms associated with transcripts showing at least one type of splicing change in primary DMSXL astrocytes (single or multiple exon skipping, mutually exclusive exons, switch of acceptor or donor sites). A maximum of five terms are shown per ontology (biological process, cellular component and molecular function), in decreasing order of enrichment ratio.

GO code	Term	Enrichment ratio	FDR
**Biological process**			
GO:0051646	Mitochondrion localization	8.7	1.3E-02
GO:0031122	Cytoplasmic microtubule organization	6.4	2.8E-02
GO:0035418	Protein localization to synapse	5.9	1.1E-02
GO:0099072	Regulation of postsynaptic membrane neurotransmitter receptor levels	5.2	1.5E-03
GO:0030048	Actin filament-based movement	4.8	2.2E-02
**Cellular component**			
GO:0097386	Glial cell projection	8.2	1.6E-02
GO:0005905	Clathrin-coated pit	6.3	2.3E-03
GO:0048786	Presynaptic active zone	3.9	3.8E-02
GO:0030055	Cell substrate junction	3.8	4.7E-03
GO:0005874	Microtubule	3.5	6.2E-05
**Molecular function**			
GO:0071814	Protein-lipid complex binding	11.3	1.8E-02
GO:0019894	Kinesin binding	8.5	4.1E-03
GO:0030165	PDZ domain binding	5.7	1.1E-03
GO:0017124	SH3 domain binding	4.7	1.8E-02
GO:0050839	Cell adhesion molecule binding	3.8	1.2E-03

Supplementary Table 5. Gene ontology analysis of proteins differentially phosphorylated in DMSXL astrocytes. The most highly enriched, non-redundant, significant terms associated with proteins exhibiting phosphorylation changes in at least one phosphosite in primary DMSXL astrocytes. A maximum of five terms are shown per ontology (biological process, cellular component and molecular function), in decreasing order of enrichment ratio.

GO code	Term	Enrichment ratio	FDR
Biological process			
GO:0030010	Establishment of cell polarity	4.9	3.3E-03
GO:0000902	Cell morphogenesis	2.3	3.3E-03
GO:0007010	Cytoskeleton organization	2.1	3.3E-03
GO:0048869	Cellular developmental process	1.6	9.7E-03
GO:0023052	Signaling	1.5	9.7E-03
Cellular component			
GO:0005913	Astrocyte projection	9.9	3.3E-02
GO:0005912	Cell-cell adherens junction	6.5	2.6E-06
GO:0097458	Intermediate filament cytoskeleton	4.3	3.3E-02
GO:0044456	Cortical actin cytoskeleton	4.1	2.1E-02
GO:0098590	Clathrin-coated pit	3.8	3.2E-02
Molecular function			
GO:0003779	Actin binding	2.7	5.4E-03
GO:0017022	Myosin binding	5.0	1.3E-02
GO:0051020	GTPase binding	2.2	1.8E-02
GO:0030551	Cyclic nucleotide binding	13.7	3.8E-02
GO:0015631	Tubulin binding	2.6	3.9E-02

2.2 Supplementary Figures

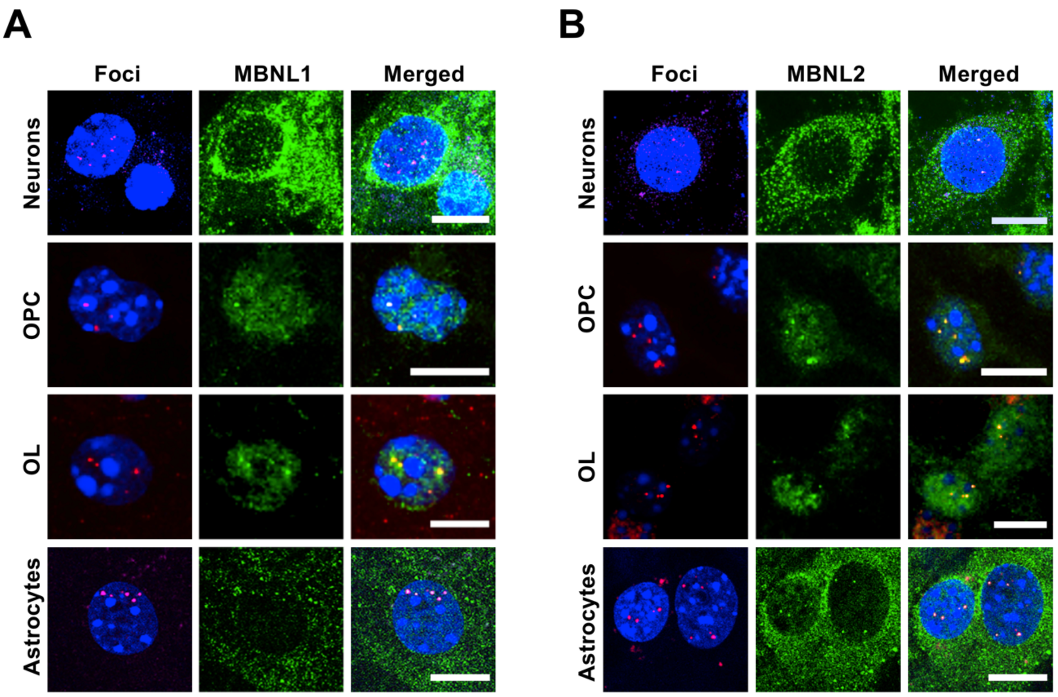

Supplementary Figure 1. MBNL co-localization with nuclear RNA foci in primary DMSXL mouse brain cells. Representative images of neurons, OPC, OL and astrocytes showing the co-localization of RNA foci with either MBNL1 **(A)** or MBNL2 **(B)**. Scale bars represent 10 µm.

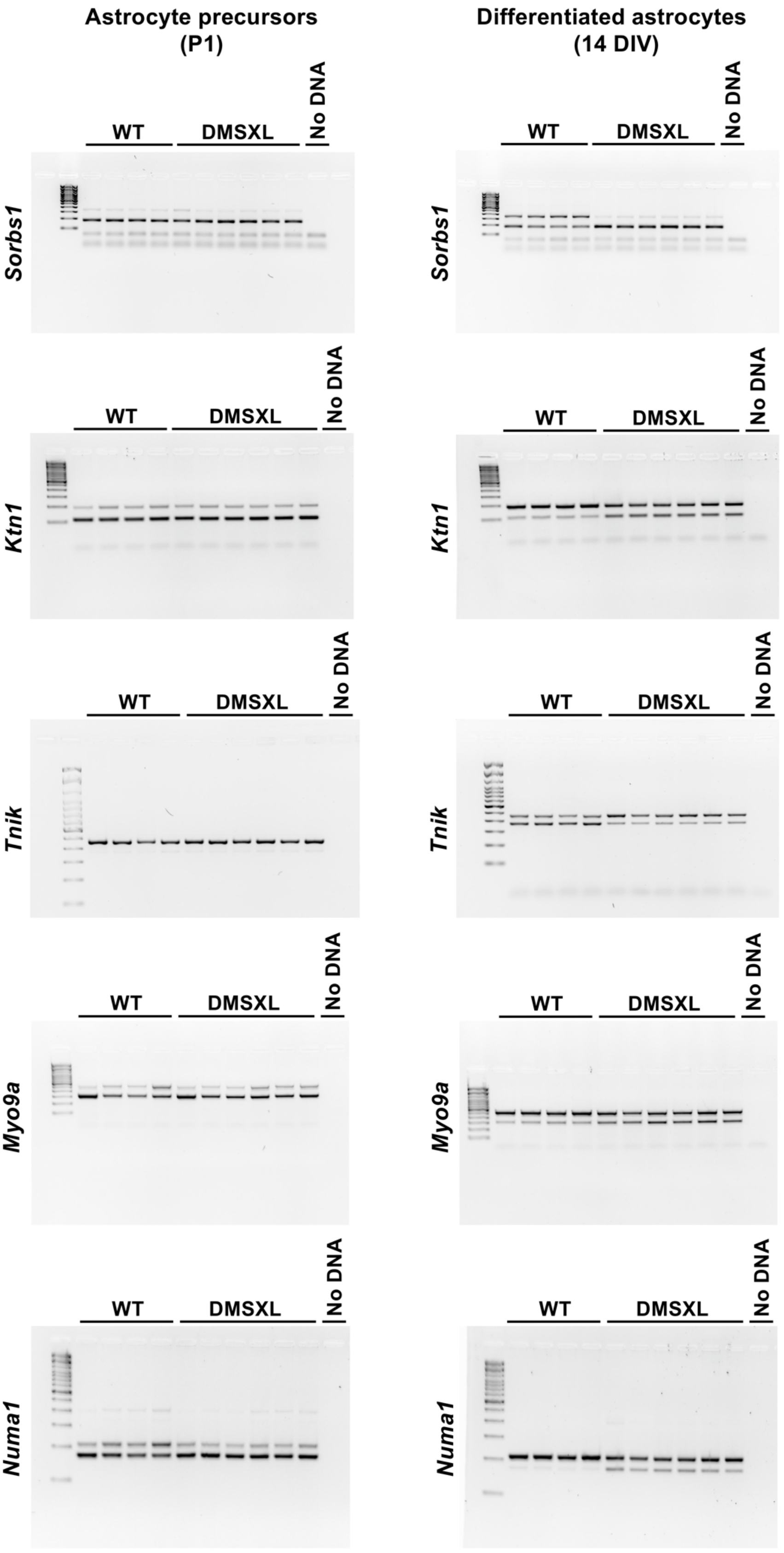
Astrocyte precursors
(P1)
Differentiated astrocytes
(14 DIV)
WT
DMSXL
No DNA
Sorbs1
Ktn1
Tnik
Myo9a
Numa1

Supplementary Figure 2. RT-PCR analysis of alternative splicing in primary DMSXL astrocytes. Uncropped images used to prepare Figure 5. Image display inverted for better visualization. MW Ladder: GeneRuler 100 bp DNA Ladder (Thermo Fisher, SM0244).

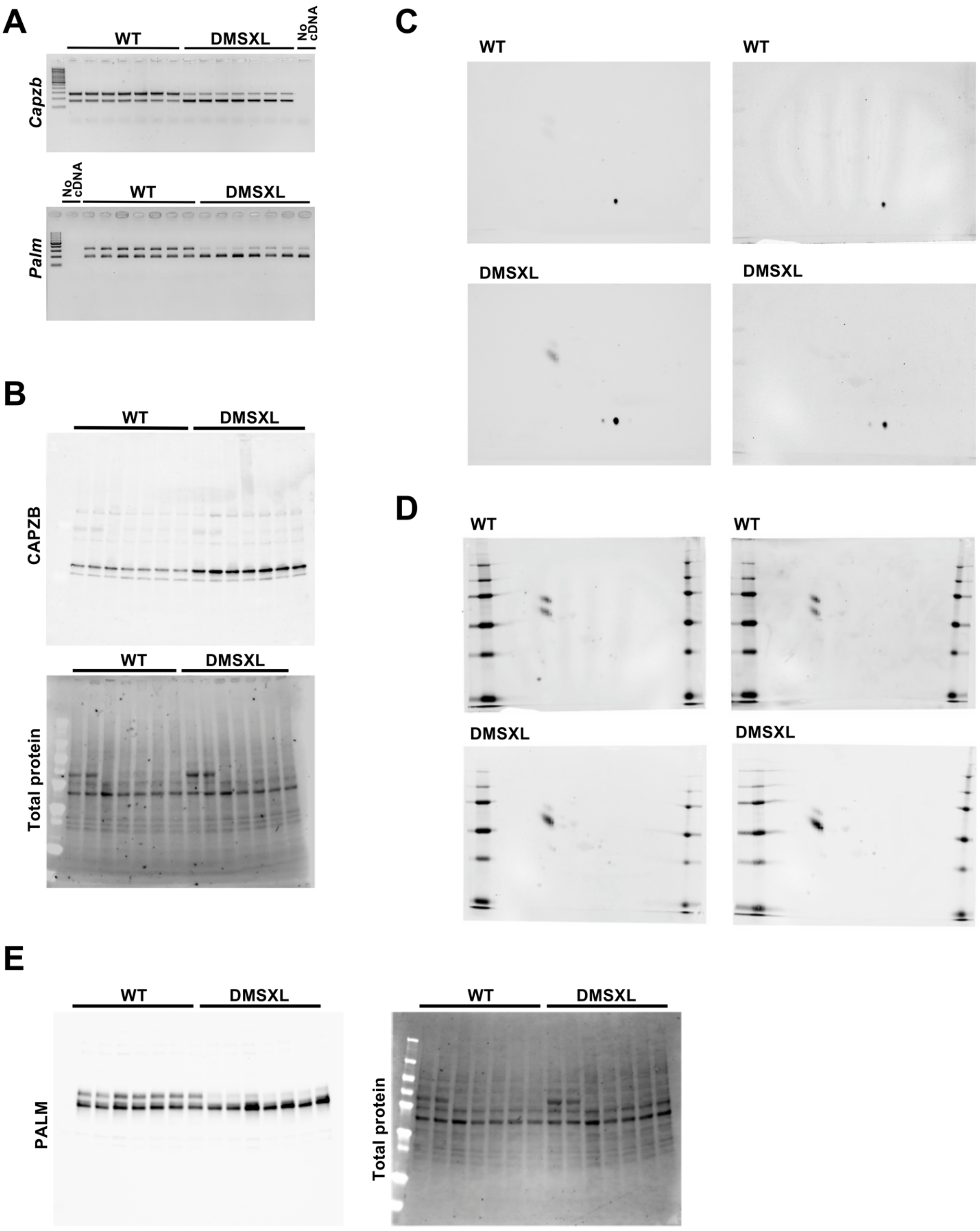
A
WT
DMSXL
No cDNA
Capzb
No cDNA
WT
DMSXL
Palm
B
WT
DMSXL
CAPZB
WT
DMSXL
Total protein
C
WT
WT
DMSXL
DMSXL
D
WT
WT
DMSXL
DMSXL
E
WT
DMSXL
PALM
WT
DMSXL
Total protein

Supplementary Figure 3. Analysis of splicing, phosphorylation and total protein levels of CAPZB and PALM in DMSXL astrocytes. Uncropped images used to prepare Figure 7. **(A)** Images of RT-PCR splicing analysis of mouse *Capzb* and *Palm* transcripts in primary mouse astrocytes. Image display inverted for better visualization. MW Ladder: GeneRuler 100 bp DNA Ladder (Thermo Fisher, SM0244). **(B)** Images of CAPZB western blot detection and total protein. MW Ladder: PageRuler Plus Prestained Protein Ladder (Thermo Scientific, 26619). **(C)** Images of CAPZB two-dimension western blot. Image display inverted for better visualization. MW Ladder: Precision Plus All Blue (Bio-Rad, 161-0373). **(D)** Images of PALM two-dimension western blot. Image display inverted for better visualization. MW Ladder: Precision Plus All Blue (Bio-Rad, 161-0373). **(E)** Images of PALM western blot detection and total protein. MW Ladder: PageRuler Plus Prestained Protein Ladder (Thermo Scientific, 26619).

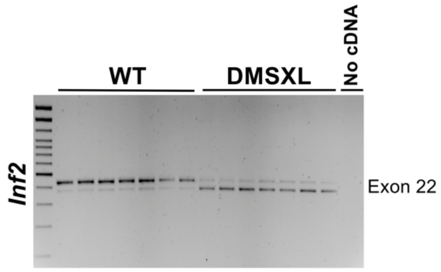

Supplementary Figure 4. Splicing analysis of *Inf2* in DMSXL astrocytes. Uncropped image of gel electrophoresis analysis of mouse *Inf2* isoforms in WT and DMSXL astrocytes (n = 7 independent cultures, per genotype). Alternative exon is indicated on the right. Image display inverted for better visualization. MW Ladder: GeneRuler 100 bp DNA Ladder (Thermo Fisher, SM0244).

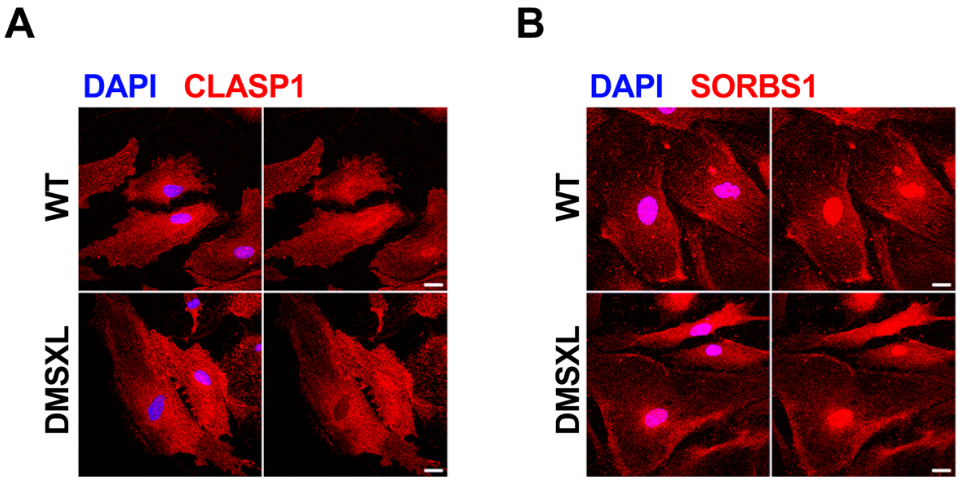

Supplementary Figure 5. Localization of CLASP1 and SORBS1 proteins in DMSXL astrocytes. Representative confocal images of the localization of **(A)** CLASP1 and **(B)** SORBS1 proteins in primary DMSXL and WT astrocytes. No obvious difference in protein intracellular distribution was detected between the two genotypes. Scale bars represent 20 µm.
